# Genetic interaction networks mediate individual statin drug response in *Saccharomyces cerevisiae*

**DOI:** 10.1038/s41540-019-0112-5

**Published:** 2019-10-03

**Authors:** Bede P. Busby, Eliatan Niktab, Christina A. Roberts, Jeffrey P. Sheridan, Namal V. Coorey, Dinindu S. Senanayake, Lisa M. Connor, Andrew B. Munkacsi, Paul H. Atkinson

**Affiliations:** 1 Centre for Biodiscovery, School of Biological Sciences, Victoria University of Wellington, Wellington, New Zealand; 2 European Molecular Biology Laboratory, Meyerhofstraße 1, 69117 Heidelberg, Germany

**Keywords:** Epistasis, Biochemical networks, Screening, Epistasis

## Abstract

Eukaryotic genetic interaction networks (GINs) are extensively described in the *Saccharomyces cerevisiae* S288C model using deletion libraries, yet being limited to this one genetic background, not informative to individual drug response. Here we created deletion libraries in three additional genetic backgrounds. Statin response was probed with five queries against four genetic backgrounds. The 20 resultant GINs representing drug–gene and gene–gene interactions were not conserved by functional enrichment, hierarchical clustering, and topology-based community partitioning. An unfolded protein response (UPR) community exhibited genetic background variation including different betweenness genes that were network bottlenecks, and we experimentally validated this UPR community via measurements of the UPR that were differentially activated and regulated in statin-resistant strains relative to the statin-sensitive S288C background. These network analyses by topology and function provide insight into the complexity of drug response influenced by genetic background.

## Genetic interaction networks underlie statin efficacy

Genes are wired together in functional genetic interaction networks (GINs) specific for different traits. We asked a simple question: do GINs for particular traits vary with individuals? The trait we investigated was response to statins, cholesterol-lowering drugs prescribed to 30 million people worldwide. Building comprehensive GINs requires a library of cells with a different gene deleted covering the entire genome that is available only in *Saccharomyces cerevisiae* (Baker’s yeast), a well-defined model for human genetics. However, the yeast libraries being limited to one genetic background are not informative of individuals. Therefore, we constructed GINs in additional genetic backgrounds and showed statin-specific GINs were not conserved, albeit there was a common fundamental process mediating statin-resistance. Our results identify a mechanism to further investigate in the millions of people that do not respond to statins and the methodology will enhance the discovery and development of drugs to treat other major diseases.

## Introduction

Understanding phenotypes that are genetically complex requires analysis of multiple genes contributing to phenotypes. Classical genetic studies have long investigated the additive functions of genes in polygenic or quantitative traits,^1,2^ the effects of modifying alleles,^3,4^ genetic capacitators,^5^ “missing heritability”,^6^ the epistatic contribution to phenotype,^7^ as well as both additive and epistatic effects.^8^ In an advance, using systems biology, gene functions have also been implied in genetic interaction networks (GINs) comprising overlapping synthetic lethal genetic interactions (GIs) between pairs of non-essential gene deletions identified in high throughput by synthetic genetic array (SGA) technology.^9^ Epistasis occurs because some gene pairs have functional commonality and, logically, specific GINs of overlapping epistatic gene pairs can also be considered functional.^10–12^ We thus address the questions: are there specific functional GINs that are conserved or do these GINs vary in individuals?

The debate is open^13,14^ as the conservation and effects of perturbation of these GINs remain largely predictive^15^ via comparisons of specific synthetic lethal GIs in human and yeast,^16^ GIs in the distantly related yeasts and *Caenorhabditis elegans*,^17,18^ essential genes in two strains of *Saccharomyces cerevisiae*,^4^ and protein–protein interactions in *Schizosaccharomyces pombe* and *S. cerevisiae*.^19^ There is broad concurrence that specific genes of GINs are not well conserved but that some GIs and network topological features are conserved.^11^ However, GINs have not been well-studied with respect to individual drug response in different genetic backgrounds. Statins target HMG-CoA-reductase^20^ and are amongst the most prescribed of all therapeutic drugs.^21^ Statins inhibit the mevalonate pathway in its four main branches that besides cholesterol reduction also affect dolichol synthesis, ubiquinones and isoprenoids.^22,23^ Therefore, statin effects are quite diverse with significant side-effects in some individuals such as muscular myopathies^24,25^ and individual variation in clinical efficacy.^26^ Atorvastatin and cerivastatin are two cholesterol-lowering drugs that have the same target in the mevalonate pathway, HMG-CoA reductase (encoded by *HMGCR* in humans and the orthologous paralogues *HMG1* and *HMG2* in yeast), but cerivastatin is no longer FDA-approved owing to adverse side effects,^25^ suggesting that comparison of GINs in response to these drugs in different individual genetic backgrounds could be helpful in fully understanding the mechanisms of these drugs. The statin drug target and sterol pathways are conserved from yeast to humans,^27^ the *HMG1/2* deletion has been used as a genetic mimic of statin treatment,^22,23^ chemical genomic analyses in yeast elucidated the cellular response to statins,^28^ and genome-wide analyses in yeast identified GIs with the *HMG1/2* statin drug target.^12^

Deletion libraries in the yeast S288C background used in SGA and chemical genetic analyses have provided much of what is known about GINs in eukaryotes.^9,12,29,30^ In this paper, we extended this knowledge by creating three new deletion libraries in three additional yeast strains of different genetic backgrounds. We identified GIs with atorvastatin, cerivastatin, the statin target *HMG1*, its functional paralog *HMG2*, and the sterol homoeostatic *ARV1* in four genetic backgrounds that generated 20 GINs. Although atorvastatin and cerivastatin share the same drug target, we show here that the statins had highly variable GINs in individual strains by multiple criteria including functional, topological and clustering comparisons. Notably, the unfolded protein response (UPR) was identified as a significant response of the network topology to statins, and we functionally translated this result with experiments showing that activation of the UPR was variable depending on the statin drug and the genetic background. Network topological analysis of genes related to the UPR showed their importance particularly in community partitions. Overall, our results provide functional insight derived from topological analyses of GINs that model the complexity of GINs underlying individual drug response.

## Results

### Selection of statin-resistant strains

**Fig. 1:**
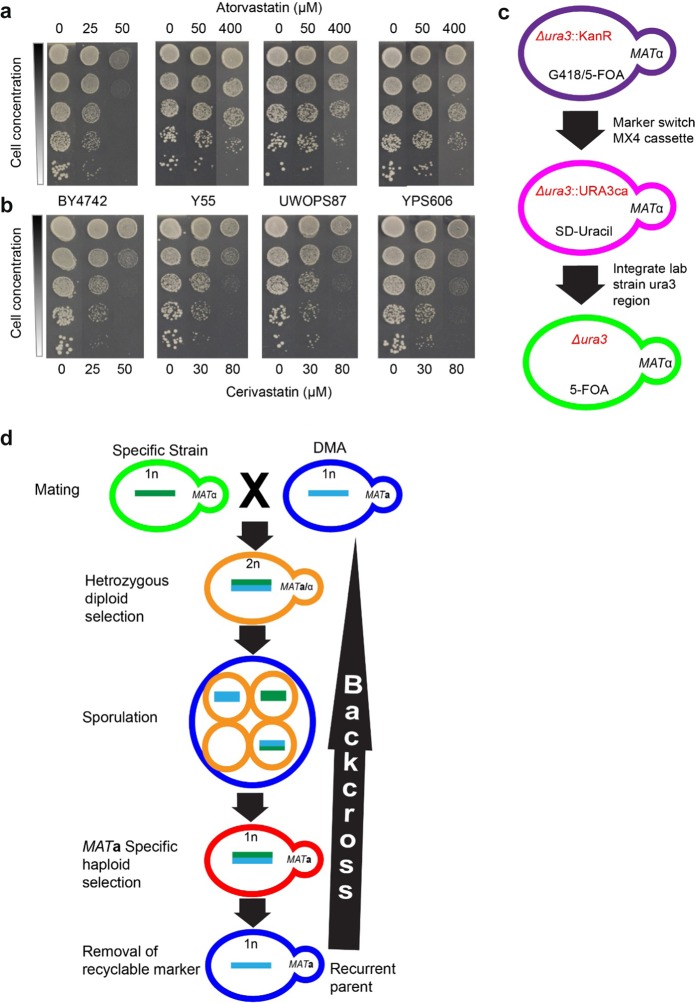
Statin resistance of SGRP strains and backcross methodology to construct ssDMA libraries that represent genome-wide deletion libraries of these SGRP strains. **a**, **b** Serial dilutions of three SGRP strains (Y55, UWOPS87-2421 and YPS606) resistant to **a** atorvastatin and **b** cerivastatin relative to BY4742 in the S288C background. **c** Outline of marker switch method used to introduce the *URA3* marker into the SGRP strains. **d** Outline of the method used to cross and backcross the SGRP strains with the S288C deletion library (DMA) to produce the ssDMA libraries

We obtained 36 haploid derivatives (Supplementary Table S1) from the fully sequenced wild-type strains in the Saccharomyces Genome Resequencing Project (SGRP) collection^31^ and evaluated growth (growth was used as an end point fitness measurement unless stated otherwise) via serial spot dilutions on agar plates containing increasing concentrations of atorvastatin or cerivastatin. There was a large range of phenotypic diversity observed for these strains with some being ∼10-fold more sensitive and others ∼10-fold more resistant relative to BY4742 (the wild-type strain in the S288C background). Three strains (Y55, UWOPS87-2421 and YPS606) exhibited normal growth at 400 μM atorvastatin in contrast to 50 μM atorvastatin that was lethal to S288C (Fig. 1a). Likewise, growth of these strains at 80 μM cerivastatin was comparable with growth of S288C at 50 μM cerivastatin (Fig. 1b). These strains were chosen for further investigation.

### Construction and validation of three new strain deletion mutant arrays

To investigate GINs underlying resistance of these strains to statins, we constructed new strain-specific genome-wide deletion libraries for the three statin-resistant strains (Y55, UWOPS87-2421 and YPS606) for subsequent use in chemical genetic and SGA analyses. First, we used a marker-switching strategy to introduce *ura3-Δ* and *his3-Δ* into the SGRP strains (Fig. 1c, Supplementary Table S1). Then we used selection markers within the SGA methodology^30^ to cross and backcross these strains six times with the S288C deletion library (Fig. 1d). The SGRP strains for making these libraries had sporulation rates of 70–80% compared to only 3% in S288C, hence greatly facilitating the sporulation step in SGAs with the new deletion libraries. We named the new deletion libraries SGRP strain deletion mutant arrays (ssDMAs), which retained the statin-resistant phenotype (Supplementary Fig. S1). Whole-genome sequencing of the new strains was performed from 384 pooled colonies of a specific plate (plate 10) from each of the three ssDMAs and the S288C deletion library, which were further compared with the S288C haploid derivative BY4742. Each ssDMA sequence had a 0.2–0.4% higher alignment score to its SGRP parental strain than to the BY4742 parental strain (Supplementary Table S2). Furthermore, each ssDMA sequence had more regions with perfect (100%) alignment with its UWOPS87-2421, Y55 and YPS606 SGRP parent strains than the S288C parent (Supplementary Figs. S2–S4), confirming that the background of each ssDMA closely resembled its SGRP parent. Synteny analysis showed that there were not any major structural rearrangements in the ssDMA or parental SGRP strains (Supplementary Figs. S5–S7). Synteny with S288C was also supported with the recovery of the expected linkage groups surrounding the three query genes in the SGAs later described (Supplementary Table S3). Analysis of the sequencing coverage indicated that each ssDMA was euploid, consistent with the controls (Supplementary Fig. S8). Genetic variation between the strains was also evident revealed by 5.9, 5.4 and 5.9 SNPs/kb in Y55, UWOPS87-2421 and YPS606, respectively, a finding that generally exceeded the SNP density among ethnically distinct human populations,^32^ thus indicating there is extensive genetic diversity in our yeast backgrounds to investigate genetic background effects on GINs.

### Chemical genetic and GI profiles differ between yeast strain strains

**Fig. 2:**
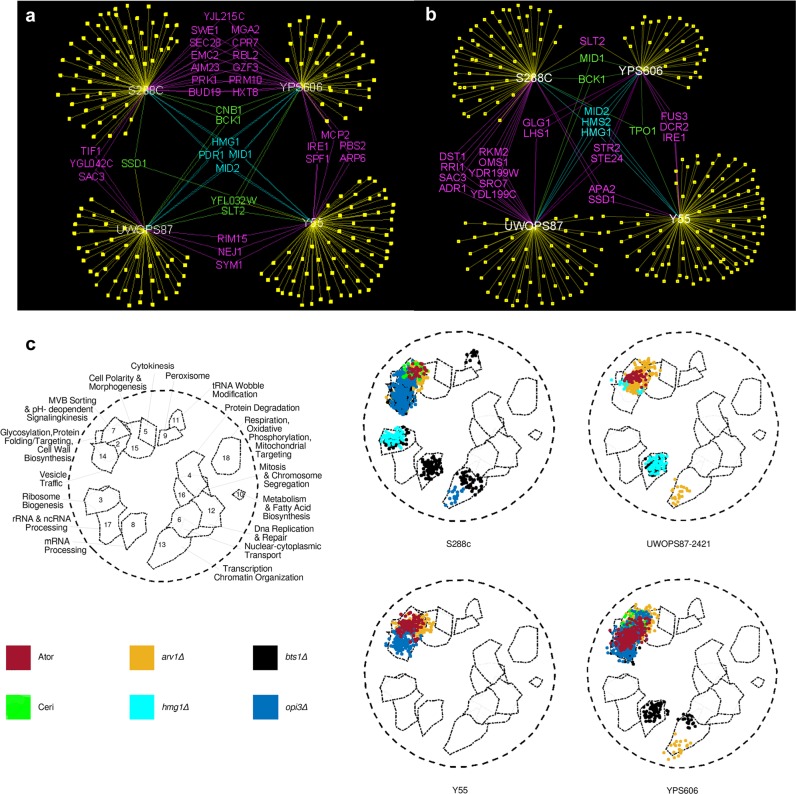
Primary genetic interaction networks and functional annotation illustrates variation across genetic backgrounds. **a**, **b** Chemical genetic profile for atorvastatin (**a**) and cerivastatin (**b**) showing interactions shared by different genetic backgrounds (purple, green and blue) or interactions unique to specific genetic backgrounds (yellow, genes not included in this figure). **c** Spatial analysis of functional enrichment (SAFE) of 20 GINs derived from five queries (atorvastatin, cerivastatin, *hmg1*-*Δ*, *hmg2-Δ*, *arv1-Δ*) and four genetic backgrounds (S288C, UWOPS87-2421, Y55, YPS606). Relative to the 18 functional regions previously defined,^13^ the figure illustrates significant membership of each GIN to these functional regions where the intensity of the colour is proportional to the number of interactions

The central tenet of chemical genetics^33^ is that a drug binds specifically to a gene product, alters or ablates its function, and mimics a mutation. Thus, a drug can be paired with a deletion mutation to screen for hypersensitive epistatic interaction profiles en masse in deletion libraries to provide information on the target and buffering mechanisms of the drug.^29^ We obtained chemical genetic profiles of our four deletion libraries via growth measurements in the presence and absence of statin treatment (25 µM atorvastatin and 10 µM cerivastatin for the S288C DMA, 100 µM atorvastatin and 50 µM cerivastatin for the statin-resistant ssDMAs). These concentrations inhibited the susceptible and resistant strain growth to approximately the same amount (Fig. 1a). The chemical genetic profiles for atorvastatin (Fig. 2a) and cerivastatin (Fig. 2b) were substantially different in the four strains. In the profile of 288 gene deletions hypersensitive to atorvastatin, only four deletions (*HMG1, MID1, MID2, PDR1*) were common to all four strains. In the profile of 283 gene deletions hypersensitive to cerivastatin, only three gene deletions (*HMG1, HMS2, MID2*) were common in all four strains. There was a requirement for *SPF1* in the resistant strains YPS606 and Y55 in the presence of atorvastatin and cerivastatin, but not in S288C. Interestingly, *SPF1* is an endoplasmic reticulum (ER) ATPase ion transporter that regulates *HMG2* degradation^34^ and interacts with the UPR regulators *HAC1* and *IRE1*,^35^ implicating buffering by the ER UPR system in the statin drug response.

The overall profiles were distinct in the different strains because most of the hypersensitive gene deletions were unique to one strain (Supplementary Table S4). Only one interaction for atorvastatin was common to the three resistant strains and not S288C; this was *SLT2*, a MAP kinase regulator of proteasome abundance that has GIs with the major protein folding sensors *IRE1* and *HAC1*^35^ as well as with *CWH41*, a glycosylation processing glucosidase critical to sensing protein folding in the ER.^36^ In yeast, the major mediator of the UPR is *IRE1*, which upon ER unfolded protein stress, is released from the Hsp70 chaperone *KAR2* in the ER whence it oligomerises and activates the UPR pathway.^37^ The *ire1-Δ* strain was hypersensitive to atorvastatin and cerivastatin in Y55 and YPS606 (Fig. 2a, b). In contrast, IRE1 was not required in S288C to buffer the effect of atorvastatin or cerivastatin.

We next performed strain-specific SGAs utilising the query strains *hmg1-Δ*, *hmg2-Δ* and *arv1Δ*, since all three genes are fundamental to sterol homoeostasis in the ER, synthetic lethal with *IRE1*^38–40^ or involved in glycosylphosphatidylinositol (GPI) biosynthesis, which is one of the major branches of the mevalonate pathway. The 12 SGA procedures generated 50,400 unique double deletion mutants, of which 37,800 have not been previously constructed. The negative GIs (synthetic lethal and synthetic sick interactions) for each background were scored as double deletion strains (*Z*-score > 2.0, *P*-value < 0.05) with significant growth defects in the double mutants relative to the single deletion mutants. Genes in the linkage groups of the *hmg1-Δ*, *hmg2-Δ* and *arv1-Δ* queries were excluded as GIs (Supplementary Table S3). Across the four genetic backgrounds, 339 GIs were identified with the *hmg1-Δ* query, 181 GIs with the *hmg2-Δ* query and 425 GIs with the *arv1-Δ* query (Supplementary Table S4; Supplementary Figs. S9–S11). Overall, there was only limited overlap of GIs for each query strain across the four genetic backgrounds. Specifically, the *hmg1-Δ* query gene exhibited overlapping GIs with *hmg2-Δ* in all backgrounds (Supplementary Fig. S9), GIs unique to the resistant strains (*e.g*., *SEC66, HTZ1, NUM1, YML102C, SHE4* and *IRE1*) were common to two resistant strains Y55 and UWOPS87-2421, and GIs shared by sensitive and resistant backgrounds (*SAC1, SPF1* and *GIM5*) were common to the sensitive S288C as well as the resistant Y55 and UWOPS87-2421. For the *hmg2-Δ* query, only *HMG1* was a common GI to all four strains (Supplementary Fig. S10) and the helicase *YRF1-6* was common to two resistant strains (Y55, UWOPS87-2421). The *arv1-Δ* query gene exhibited GIs only with *SLT2, CDC73, CHS3, CHS7* and *IRE1* in all four strains, while GIs shared by only the resistant strains were with *LEO1, YBL062W, RPS8A, SKT5* and *HMG1* (Supplementary Fig. S11). The various GIs arising from the statin chemical genetic profiles and the SGA queries indicated, among other processes, a clear involvement of early secretory pathway processes.

### SAFE analysis reveals functional heterogeneity of gene usage among yeast strains

We performed spatial analysis of functional enrichment (SAFE) analysis^10,12^ to elucidate the functional properties of the GINs. SAFE is a Gene Ontology (GO) functional depiction analysis with network topology constrained graph distance similarities that represents statistically and quantitatively localised enrichments of genes in specific cellular processes.^10^ The atorvastatin treatment shows a concentration of GIs enriched in the early secretory pathway related Region 2 (glycosylation/protein folding) and Region 7 (multivesicular body (MVB) sorting) in all four strains (Fig. 2c; Supplementary Table S5). Notably different to the other strains, the resistant strain YPS606 was also significantly enriched in Region 14 (vesicle trafficking). In contrast, the cerivastatin treatment showed a concentration of GIs enriched in Region 2 (glycosylation/protein folding) and Region 7 (MVB sorting) only in S288C and Y55, compared to Region 15 (cell polarity and morphogenesis) only in S288C. These results show that not only do the strains differ functionally but that the cerivastatin response is markedly different to atorvastatin. Consistent with the SAFE chemical genetic profiles, SAFE analyses of GIs with the query genes *HMG1, HMG2* and *ARV1* also showed extensive heterogeneity of gene usage across the four genetic backgrounds. The GIs with *ARV1* were enriched only in Region 7 (MVB sorting) in all four strains, while there was enrichment in Region 2 (glycosylation/protein folding) and Region 14 (vesicle trafficking) in two resistant strains (YPS606 and UWOPS87-2421). In contrast, the statin-sensitive S288C GIs were concentrated in Region 14 (vesicle trafficking). Unique to YPS606 was an enrichment in Region 13 (transcription, chromatin organisation). The GIs with *HMG1* and *HMG2* were enriched in Region 3 (ribosome biogenesis) in only two strains (S288C and UWOPS87-2421) with a strikingly unique signature in Region 8 (mRNA processing) in the UWOPS87-2421 strain.

### Functional hierarchical clustering shows extensive strain variation

**Fig. 3:**
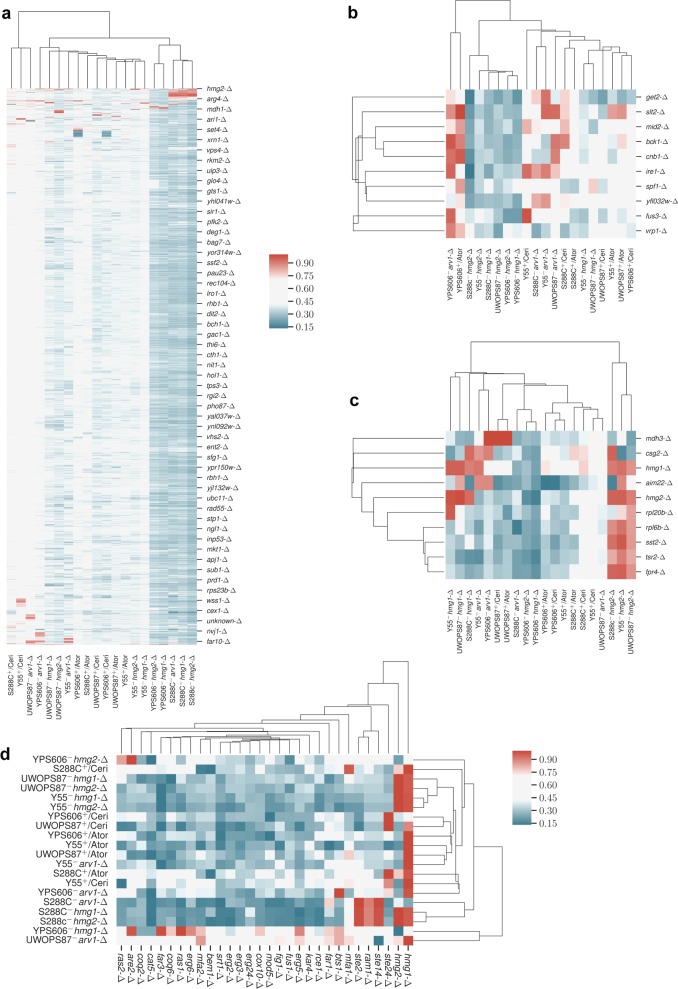
Hierarchical clustering of GINs represents overall genetic background variation throughout the genome and specifically the unfolded protein response and mevalonate pathway. **a–d** Hierarchical clustering of growth phenotypes showing **a** genome-wide deletion libraries, **b** *IRE1* (the major mediator of UPR), **c** *HMG1* and *HMG2* (the targets of atorvastatin and cerivastatin), and **d** genes within and branching from the mevalonate pathway. One scale applies to **a–c** and the scale for **d** shown separately to the right of the cluster. All scales represent min–max normalised *Z*-scores

We next elucidated functional similarities among chemical genetic and GI profiles by employing agglomerative and *k*-means hierarchical clustering^41^ based on *Z*-scores. We distinguished three major clades (Fig. 3a). The first clade had five GI profiles; these were GI profiles only in the S288C and YPS606 strains (GIs with *HMG1* and *HMG2* in YPS606 as well as GIs with *HMG1*, *HMG2* and *ARV1* in S288C). The second clade comprised six chemical genetic profiles (particularly the atorvastatin chemical genetic profile in all four backgrounds but only the cerivastatin chemical genetic profile in UWOPS87-2421 and YPS606) and six GI profiles (GIs with *ARV1* in Y55 and YPS606, GIs with *HMG1* in Y55 and UWOPS87-2421, and GIs with *HMG2* in Y55 and UWOPS87-2421). The third clade comprised two chemical genetic profiles (cerivastatin in S288C and Y55) and the GIs with *ARV1* in UWOPS87-2421. These results indicate that S288C is functionally more closely related to YPS606, and Y55 is more closely related to UWOPS87-2421, while Y55 and UWOPS87-2421 were markedly different. We narrowed the focus of Fig. 3a to ten contiguous genes re-clustering around *IRE1* the major mediator of UPR. Consistent with clustering of the whole genome, there was clear evidence of strain-dependent clustering of genes such as clusters focussed around *IRE1* (Fig. 3b) from the *hmg2-∆* query in S288C, Y55 and UWOPS87-2421 strains that were distinct from the *hmg2-∆* query in YPS606 (Fig. 3b). *IRE1* clustered closely with *SPF1* across all backgrounds (Fig. 3b), reiterating the primordial association of sterol/lipid homoeostasis and the UPR. *SPF1* is an ER membrane protein regulated by the UPR^42^ that is synthetic lethal with *BTS1*, a major branch point in the mevalonate pathway*. SPF1* is required for regulating Hmg2p degradation,^22,23^ it is involved in membrane sterol homoeostasis,^43^ and is an important functional hub interacting with many genes of the ER folding pathways, the mevalonate pathway and the early secretory pathway.

In a second focussed clustering analysis around the statin target *HMG1*, strain-dependency was again detected. *HMG1* clustered strongly with *CSG2* (an ER protein involved in sphingolipid mannosylation that has GI with *HMG1*) in the S288C/*hmg2-∆*, S288C/*hmg1-∆*, Y55/*arv1-∆* queries and to a lesser extent in S88C + atorvastatin S288C + cerivastatin (Fig. 3c).

A third focussed clustering analysis was based on relationship to a panel of genes curated from the mevalonate pathway and three main branches namely the ergosterol branch, dolichol *N*-glycan synthesis branch, and the all-trans GGPP isoprenoid branch of protein prenylation (Supplementary Table S4). In these clusters (Fig. 3d), one clade contained GIs with *ARV1* in the background of UWOPS87-2421 and *HMG1* in the background of YPS606, similarly to that seen in Fig. 3a. For the query genes *hmg1-∆, hmg2-∆* and *arv1-∆* in the S288C genetic background, there was a cluster containing GIs with *STE14* (ER protein mediating processing of alpha-factor and RAS proteins), *RAM1* (subunit of the CAAX farnesyltransferase in the protein prenylation branch) and *STE2* (alpha-factor pheromone receptor G-protein). These three genes are directly related to the isoprenoid branch of the mevalonate pathway.^35^ Thus, this small cluster identifies a significant dependence on the isoprenoid enzyme CAAX-farnesyltransferase after deletion of *HMG1, HMG2* and *ARV1* only in the S288C background.

### Topology of GINs partitioned by community analysis identifies functional modules

**Fig. 4:**
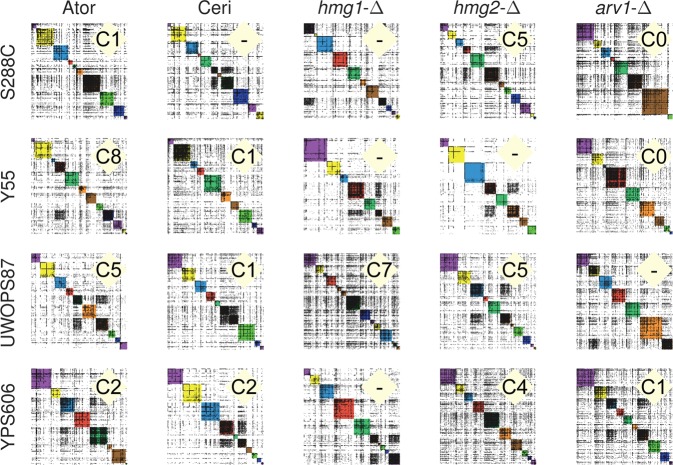
Community analysis reveals genetic background variation among GINs. Communities in each GIN were defined using community analysis described in the text. Communities shown in different colours (for visual distinction only) on the diagonal are numbered from zero at the top left where each GIN consists of between 7 and 14 communities (Supplementary Table S8). For example, the GIN in the first panel shows 10 communities. Community panels are labelled with community number (“C#”) denoting presence of the high ER/Golgi GO-term pattern described in the text

Biological gene networks are generally modular to achieve cellular function and may be defined by communities, such as partitioning of network topology based on density of node linkage patterns into modules that may reveal functional information that is not otherwise obvious.^44,45^ To perform community analysis, we augmented our primary GINs to include adjacent genes, including some essential genes, from a comprehensive yeast GIN^12^ to give greater coverage of interactions (Supplementary Table S6). We then used the Louvain^46^ community detection method, established for social networks^47^ and identified 200 communities overall in our five probe (atorvastatin, cerivastatin, *hmg1-∆, hmg2-∆* and *arv1-∆*) by four genetic background (S288C, UWOPS87-2421, Y55, YPS606) dataset. The 20 panels showed marked differences in number and density of communities dependent on genetic background (Fig. 4; Supplementary Tables S7, S8).

In the community clustering method used, a particular gene is found in only one community per GIN. If the function of the gene is known, it helps to define community function. More generally, a set of known genes in a community can be evaluated for enrichment of particular GO-terms establishing function more firmly. Since SAFE analysis showed enrichment of early secretory pathway processes (Fig. 2c), we searched for enrichments in coverage of GO terms^48,49^ of the early secretory pathway^50^ in the communities of Fig. 4. Coverage of a GO-term is the fraction (%) of the total genes known for that GO-term in a given dataset and is defined with a probability of it arising by chance. In 14/20 panels, there was one unique community (labelled in Fig. 4) enriched in ‘endoplasmic reticulum’ GO term displaying high coverage (11–21%, *P* < 0.05) that was always accompanied by the GO-term ‘Golgi apparatus’ and/or ‘Golgi vesicle transport’ (4.1–7.6% coverage; *P* < 0.01) (Supplementary Table S7). This pattern often was accompanied by ‘lipid metabolic process, protein targeting, cytoplasmic vesicles’ GO-terms demonstrating a community dedicated to early secretory pathway functions in a majority of our 20 GINs. Specific genes of the early secretory pathway, glycosylation and protein folding^39,51,52^ were present in the communities that had the ‘high ER coverage/Golgi apparatus/Golgi vesicle’ GO-term pattern (Supplementary Table S9).

In further investigation of community function, *ire1-∆* in the primary GINs (Fig. 1) was hypersensitive to statins in two of the resistant strains suggesting involvement of the UPR. Therefore, we searched all 200 communities in the 20 panels (Fig. 4) for simultaneous presence of three major sensors of the UPR (*CWH41*, *IRE1* and *HAC1)*. These three genes were found together in a single community in 11 of the 20 community panels namely the atorvastatin query (in S288C, YPS606, and UWOPS87-2421), the cerivastatin query (in S288C and UWOPS87-2421), the *hmg1-∆* query (in S288C and YPS606), the *hmg2-∆* query (in S288C, YPS606, and UWOPS87-2421), and the *arv1-∆* query (in S288C) (Supplementary Table S9). In eight of the nine remaining GINs, the co-occurrence of this motif in a single community held except that one of the three genes, usually IRE1, was found in an adjacent community. These UPR communities mostly did not overlap with the high ER-coverage pattern communities described in Fig. 4 and therefore were different communities, though related, in function.

There was also genetic background dependence shown in community partitioning of another ER function, namely all seven components (EMC1–7) of the well-documented ER membrane protein complex^38,39,52^ that were only found in statin-treated S288C communities. The whole complex was not found in any single community for any query or statin probe in the resistant strains (Supplementary Table S10), pointing to the fundamental effects of genetic background, even on the uses of essential complexes.

### Topology analysis elucidates bottlenecks of networks

**Fig. 5:**
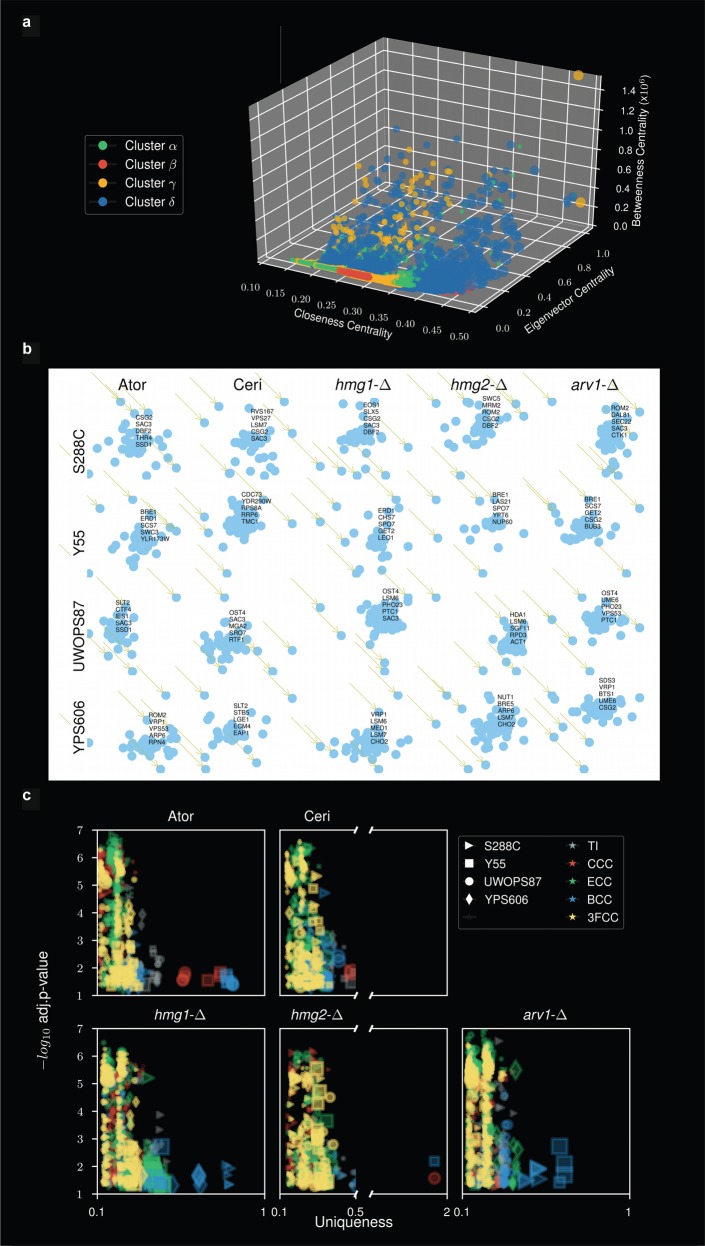
Topology analyses of augmented GINs identify betweenness bottleneck genes and functional uniqueness of GINs. **a** 3D plot of gene values calculated for closeness centrality, eigenvector centrality and betweenness centrality for the optimised four clusterings (α, β, γ, δ) of data in the augmented GINs file (Supplementary Table S6). **b** Deconvoluted driver genes with maximum betweenness centrality from atorvastatin, cerivastatin, *hmg1Δ*, *hmg2Δ* and *arv1Δ* queries in S288C, Y55, UWOPS87-2421 and YPS606. **c** Uniqueness values derived from Gene Ontology semantic analysis of driver genes using multiple topology measurements: TI (topology independent), CCC (closeness centrality cluster), ECC (eigenvector centrality cluster), BCC (betweenness centrality cluster), 3FCC (closeness, eigenvector and betweenness clusters combined). Shown here are uniqueness (a score that represents the negative of average semantic linearity of a GO term to all other terms), coverage (represented by size of the symbol) and associated statistical significance for each genetic background

Clustering by community modules is useful because membership in a community of a profile of genes may infer a function, as we have shown here. However, it is known that community methodologies suffer from scalability bias or resolution limits skewing outcomes to too few large communities or too many small communities in artificial fusions or fragmentation of natural clusters.^53–55^ Indeed, we observed community modules that occasionally showed fragmentation (e.g., cerivastatin in the YPS606 background exhibited two communities with the high ER coverage pattern/Golgi GO-term pattern (Supplementary Table S7). We therefore applied other heuristics for clustering by topological centrality terms. First, we optimised the number of centrality clusters in the augmented GIN matrices (Supplementary Table S6) using agglomerative and *k*-means clustering, testing up to ten clusters as a variable and sorting results by Silhouette (SI) clustering score^53,54^ and Calinski–Harabasz Index (C–H) score. We found between 3 and 7 clusters as optimal (Supplementary Table S11), so we processed the matrices for four clusters as a compromise calling them Cluster α, Cluster β, Cluster ϒ and Cluster δ (Table S6, columns E, F, G, H). We then analysed the content of these clusters by four centralities (betweenness centrality cluster (BCC), closeness centrality cluster (CCC), eigenvector centrality cluster (ECC) and closeness, eigenvector and betweenness clusters combined (3FCC)) comparing their values on a 3D plot. The greatest definition by centrality value was detected in the BCC genes using Cluster δ (Fig. 5a). Distributions of BCC also showed the most distinct distributions by query and genetic background (Supplementary Figs. S12–S14), thus demonstrating the fundamental importance of betweenness centrality as network-controlling bottlenecks. This relationship has previously been reported for PPI networks.^56^ We next deconvoluted the genes from the centrality distributions and identified the five genes with highest BC (Fig. 5b), CC (Supplementary Fig. S15), and EC (Supplementary Fig. S16). The BC genes are pivotal to the network integrity and as such we deem these bottleneck genes of our GINs.

To gain functional insight into the BC bottleneck genes (Fig. 5b), we used REVIGO simplification^49^ of semantically redundant GO-terms for BCC genes by a Lin’s similarity pairwise analysis for each GO term to quantify ‘uniqueness’ values^49^ in the optimised Cluster δ. Once again, betweenness as a criterion stood-out where the greatest uniqueness was derived using the betweenness centrality terms in the various query/strain combinations. The other topology centrality terms were not as well distinguished (Fig. 5c; Supplementary Table S12). Exemplified standout uniqueness values for *hmg1∆*/YPS606 and atorvastatin/UWOPS87-2421 networks were seen for mRNA processing (0.65) and ribosome processing (0.8), respectively, and the *arv1∆*/S288C network was enriched for the early secretory pathway ER-Golgi term pattern (0.55) described above in the community analysis. These terms, derived from topology analysis of augmented GINs, show marked concordance with functions revealed in the SAFE analysis (Fig. 2c) that was based on primary GINs (Fig. 2b).

### UPR activation and regulation depends on statin sensitivity

**Fig. 6:**
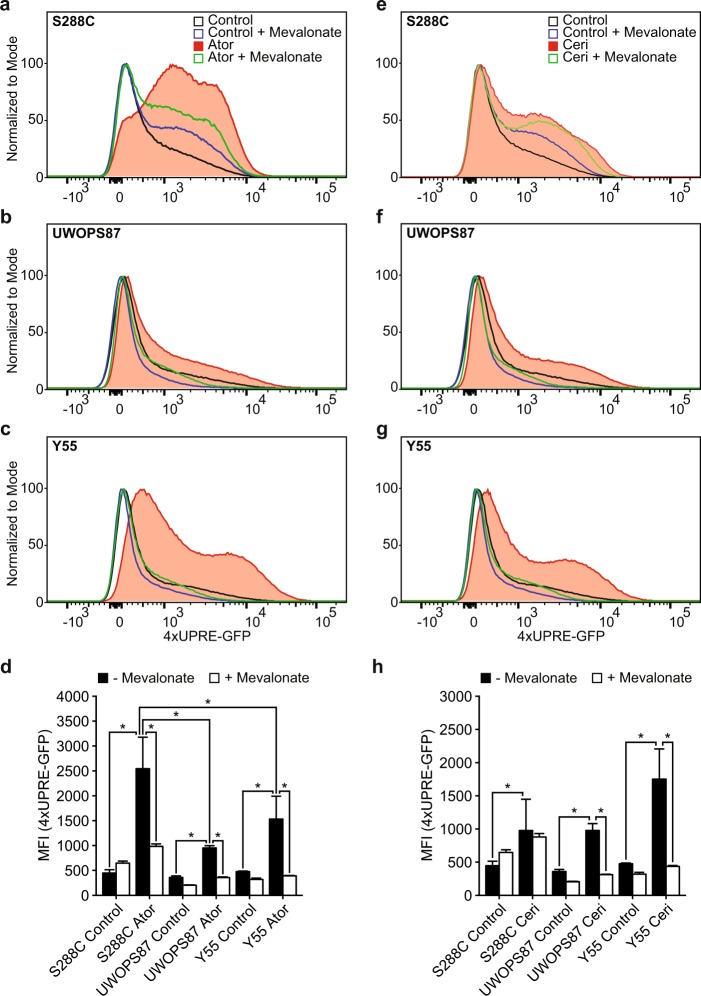
Measuring UPR activation identifies strain-specific UPR differences in response to statins. **a**–**c**, **e**–**g** Representative fluorescent distributions of triplicate data for live cells expressing a 4X-UPRE-GFP construct measured via flow cytometry and treated with or without atorvastatin (**a**–**c**) likewise cerivastatin (**e**–**g**) at IC_20_ concentrations and supplemented with or without mevalonate. **d** Mean fluorescence intensity of triplicate data for samples from **a**–**c** and **e**–**g**. Error bars represent ±standard deviation, *P < 0.05

The requirement for the major UPR sensor *IRE1* with statin treatment (Fig. 2a, b; Supplemental Table S4) in two statin-resistant strains, the enrichment of protein folding processes as a response to statin treatment (Fig. 2c), the existence of a distinct IRE1 community (Supplementary Table S9) and that S288C showed significantly more growth inhibition in DTT than the resistant strains, but not in tunicamycin (Supplementary Fig. S17A, B), suggesting a less robust UPR, led us to evaluate UPR activation in statin-sensitive and statin-resistant backgrounds. UPR induction by statins was dependent on IRE1 as determined by statin sensitivity in the four strains deleted for IRE1 (data not shown). UPR was measured via a 4X-UPRE-GFP reporter^39^ using IC_20_ concentrations of atorvastatin or cerivastatin customised for each background (0.25 µM for statin-sensitive S288C and 2.5 µM for statin-resistant backgrounds) and the readout determined by flow cytometry (gating strategy, Supplementary Fig. S18). Atorvastatin activated significantly more UPR in statin-sensitive S288C than the statin-resistant Y55 and UWOPS87-2421 (Fig. 6a–d), while there were not significant differences in UPR activation between the strains for cerivastatin treatment (Fig. 6h). To confirm the UPR is activated by inhibition of the mevalonate pathway by statins, we supplemented statin treatments with mevalonate and again measured UPR. In S288C, the UPR activated by atorvastatin was reduced with mevalonate (Fig. 6a, d) but not to control levels whilst the response to cerivastatin in S288C was not reduced at all with mevalonate (Fig. 6e, h). In the statin-resistant strains, the UPR activated by either statin was restored to control levels with mevalonate supplementation (Fig. 6b–d, f–h). Thus, UPR was entirely restored to control levels with mevalonate we termed a direct effect in the statin-resistant strains, and in contrast, the substantial UPR levels remaining above control levels after mevalonate supplementation in statin-sensitive S288C we term indirect effects of the statin treatment. We conclude that both atorvastatin and cerivastatin activated UPR differently to each other and differently in the statin-sensitive and statin-resistant genetic backgrounds.

## Discussion

This study aimed to better understand the function of GINs in different genetic backgrounds elicited by three query genes and two statin probes. For any particular trait in SGA analysis, GIs between pairs of genes indicate a functionality, which can give rise to large GINs of overlapping pairs of several hundred genes all of them potentially informative but requiring further analysis to assess a rank of importance.^45,57^ Here we investigated statin drug response by constructing GINs in different genetic backgrounds and examined these GINs by multiple criteria including functional, topological centrality, and clustering comparisons and especially by community partitioning that has not previously been conducted for GINs in yeast.

Substructure may be discerned within large network topology by modularity optimisation that partitions the network into communities of densely connected nodes within modules but sparsely connected between modules.^46^ Community detection may then allow identification of functional modules by specific enrichment of GO-terms for genes in communities allowing inference of function. Nonetheless, community analysis is an heuristic with limitations^53–55^ that depends on achievement of meaningful^45^ results for its validation.^58^ To this end, we described four unique and meaninful communities involved in ER stress; firstly, the unique communities of early ER pathway genes (Fig. 4) in response to statins and query gene deletions in all genetic backgrounds. Secondly, the EMC1-7 complex, involved in ER membrane protein insertion,^59^ folding and export,^39^ was found in specific communities only in the statin-sensitive S288C strain whilst the resistant strains did not have complete sets. The EMC complex use per se is worthy of comment because in S288C subunits individually have an effect on intracellular traffic^38^ and it is possible the number of subunits used are proportional to ER stress in S288C compared to the statin-resistant strains. Thirdly, induced ER stress requires secretory pathway gene interactions with kinetochore genes^60^ and here we detected the COMA complex (Ctf19p, Okp1p, Mcm21p and Ame1p) for atorvastatin and cerivastatin response in all genetic backgrounds (except YPS606 in cerivastatin) in one specific community (Supplementary Table S9). Fourthly, we demonstrated that UPR-specific communities containing CWH41, IRE1 and/or HAC1 had different complements of other UPR-related genes depending on genetic background and also on whether the statin drug was atorvastatin or cerivastatin. We conclude that the community heuristic we used is justified and informative of genetic background dependent communities.

Statins are known to induce UPR in the ER of *C. elegans*,^61^ in human myocytes^62^ and mouse macrophages^63^ but not in *S. cerevisiae* prior to the current study. Here we demonstrate that statins activate UPR in both statin-sensitive and statin-resistant yeast strains. Interestingly, UPR activation was restored to control levels with mevalonate only in the statin-resistant strains. This observation is possibly explainable by a direct effect on the UPR via ER protein mis-folding and ER-associated degradation (ERAD)^39,51,52^ in the resistant strains. We also found an indirect effect of mevalonate supplementation on UPR induction in the statin-sensitive S288C that arises from a quite different mechanism for induction, one mediated by CSG2 which was a significant bottleneck gene in the response to atorvastatin in the S288C genetic background. CSG2 is involved in sphingolipid synthesis,^64^ autophagy,^65^ its loss induces UPR,^39^ and it interacts extensively with the secretory pathway genes.^35^ It is required for mannosylation of inositolphosphorylceramide MIPC and M(IP)2C,^35^ and in all genetic backgrounds treated with atorvastatin and cerivastatin, CSG2 is required only in S288C. We therefore suggest that since S288C has a less robust UPR, it requires an autophagy backup when ERAD is saturated^66^ and that the indirect mevalonate effect relates to autophagy balancing ER stress,^67^ mediated by CSG2. This interpretation is supported by the requirement for a much larger number of endosomal/retromer/autophagy-associated genes in S288C than in the resistant strains in UPR communities (Supplementary Table S9). Dependence on autophagy for buffering UPR in S288C is similar to observations reported for *C. elegans*^68^ and human cells.^69^

Comparisons of GIs between different yeast species have been published,^17,18^ as well as intraspecific comparisons of quantitative trait nucleotides for sporulation efficiency that were dependent on genetic background in 13 yeast strains,^70^ comparisons of stress quantitative trait loci (QTL) that varied across four different yeast strains,^31^ comparisons of QTL for ketoconazole, benomyl response that varied across four advanced intercross yeast lines,^71^ comparisons of genes involved in conditional lethality segregating in a cross of two yeast strains,^13,14^ and comparisons of genetic modifiers of GIs that varied across genetic backgrounds.^3^ Prior to our current study that compared genome-wide GINs in four genetic backgrounds of the same species, an equivalent study had not been conducted. A similar approach was reported in bacteria in a study on antibiotic resistance,^72^ which concluded there was little conservation of GINs in response to daptomycin in two different strains of *Streptococcus pneumoniae*. Our current study extends these results with comparison of network topological centralities and communities that includes experimental validation of community analysis. We conclude that if the four yeast strains studied here can be seen as a proxy for individuals, then there is little conservation of GINs among individuals.

If specific GINs are not conserved across genetic backgrounds, then what are the functions of GINs? GINs have been described as having intrinsic functionality^73^ or as functional modules related to topological modules.^74,75^ Thus, one might ask if GINs involving hundreds of genes are units of heritability? We suggest that GINs may not be units of heritability because GINs relating to a specific phenotype, the UPR response to statins in this paper, were not conserved in different strains. Nonetheless, although GINs appear to be ephemeral or transitory, it is possible that GINs have a role in evolutionary potential.^72,76^ The concept of an evolutionary ratchet of sequentially accumulating single mutations stabilised by within-protein epistatic effects describing the evolution of the glucocorticoid receptor^77^ might be instructive. It is thus plausible that GINs emergent on a new mutation are an evolutionary ratchet that irreversibly stabilise a new mutation one at a time allowing increased fitness through transient epistatic mechanisms superseded over time by additional mutations. The GINs in this model would not be heritable but their effects would be heritable. Such a model could be tested by observing whether an extant GIN seen in a particular strain deletion library was ephemeral after additional meioses but stable after additional mitoses.

In summary, we show the statin-resistant strains were dependent on protein folding and early secretory pathway genes that were sufficient to alleviate the requirement for the mevalonate-dependent UPR. By contrast, in the statin-susceptible S288C strain, ER stress was evident with greater induction of the UPR that was both dependent and independent of mevalonate. Topological centrality analysis identified betweenness gene bottlenecks that are likely of key importance in the statin response. We conclude that GIN function and the topological properties of the network induced by a drug, in addition to the main target, should be considered in the drug mechanism of action and efficacy. The use of deletion libraries in multiple genetic backgrounds in the tractable yeast system to elucidate GINs will guide future eukaryotic chemical genomic analyses from yeast to human cells in such investigations.

## Methods

### Yeast strains and media

Strains of *S. cerevisiae* (Supplementary Table S1) including SGRP strains (National Collection of Yeast Cultures (NCYC), Norwich, United Kingdom) and the S288C DMA library were maintained in synthetic complete (SC), synthetic dropout (SD), enriched sporulation, or yeast peptone dextrose (YPD) as previously described.^78^ Media included yeast extract, peptone, yeast nitrogen base without amino acids and ammonium sulphate, amino acids and agar (Formedium), nourseothricin (Werner BioAgents), geneticin (G418; Carbosynth) and hygromycin B (HPH; Life Technologies), 5-fluoroorotic acid (Kaixuan Chemical), atorvastatin (Inter Chemical), cerivastatin (Chengdu Caikun Biological Products) and ampicillin, canavanine and *S*-aminoethyl-l-cysteine (Sigma).

### Backcross construction of SGRP deletion mutant array (ssDMA) libraries

Backcross methodology was used to construct ssDMA libraries. Appropriate selection markers were introduced in SGRP strains via PCR-mediated disruption with a variety of primers (Supplementary Table S13). The KanR marker at the *URA3* locus in SGRP strains was replaced with pAG60-derived *CaURA3* (Supplementary Table S14),^79^ which was then removed by homologous recombination with the flanking *ura3Δ0* region from BY4742, followed by growth on 5-FOA media selecting for *URA3* null mutation. The selected SGRP strains were crossed with the original S288C DMA and the hybrid progeny backcrossed five more times with the SGRP strains, resulting in three new deletion libraries we termed “ssDMAs” for SGRP strain deletion mutant arrays.

### Genome sequence analysis and genomic authenticity of ssDMA libraries

The ssDMA strains were subjected to whole-genome sequence analysis to determine the efficacy of the backcross strategy. Genomic DNA was extracted from pooled yeast strains from plate 10 of the DMA and ssDMA libraries. Truseq Nano 350 bp insert libraries were prepared using a TruSeq Rapid SBS kit or TruSeq SBS kit v4 and sequenced on an Illumina HiSeq2000 instrument (Macrogen). Raw image processing, base calling and conversion to FASTQ format was carried out at Macrogen by HiSeq Control Software v2.2, Real Time Analysis v1.18.61, and bcl2fastq v1.8.4. All sequence data were aligned to the *S. cerevisiae* S288C reference genome^35^ and SGRP strains.^80^ The data alignment pipeline was carried out as previously described.^71^ Whole Genome Vista^81^ was used to align the FASTA sequence of each ssDMA to the respective parental SGRP strain. The pairwise alignment score was calculated from the summation of the alignment score of each aligned fragment with a weighting on the basis of fragment size and filtration to only retain regions that had 100% alignment between the ssDMA sequence and parental strains. Sequence coverage in the final BAM file was used to determine if any aneuploidy was evident in any of the ssDMA libraries. Per-base sequence coverage was exported using Unipro UGENE v1.27 and the mean average coverage was calculated for each 20 kb genomic bin.

### SGA analysis

SGA analysis was conducted as previously described^30^ with the additional methodology of integrating SGA reporters in the MATα ssDMA strains. The *CEN-URA3* marker (pRS316) was expressed in the standard SGA query strain (Y7092) with the reporter (*can1Δ::STE2pr-Sp_his5*), mated with the SGRP parent, and the CEN-URA3 marker was removed with selective killing on media containing 5-FOA. Replica plating (pinning) was performed using an automated RoToR HDA system (Singer Instruments). Double deletion mutants at completion of the SGA analysis were incubated at 30 °C for 48 h and imaged using a digital camera (Canon EOS 600D).

### Chemical genetic analysis

Growth (measured as an end point after 48 h, used as a proxy for fitness) of ssDMA and S288C libraries was evaluated at concentrations of atorvastatin and cerivastatin that inhibited growth by ~40% in SC media. These concentrations of atorvastatin (25, 100 µM) and cerivastatin (10, 50 µM) were based on growth of plate 10 in each library in the screening format of 1536 colonies per plate. The S288C and ssDMA libraries were pinned in 1536 format on the media containing either atorvastatin, cerivastatin or DMSO (vehicle control), incubated at 30 °C for 48 h, and imaged using a digital camera (Canon EOS 600D).

### Phenotypic analysis

Colony size and circularity were measured using Gitter^82^ and statistically compared using ScreenMill.^83^ Growth ratios (double deletion vs single deletion, treated single deletion vs untreated single deletion) were represented in *Z*-score values that were statistically evaluated using a normal distribution. Interactions (*Z* > 2.0, *P* < 0.05) were visualised using Cytoscape v3.5.1.^84^

### SAFE analysis

SAFE analysis was performed as previously described^10^ using the global GIN of *S. cerevisiae*,^12,35^ where the map-weighted shortest path length was the distance metric, maximum distance from a node was set at 7.5 radius, and threshold for enrichment significance was 0.05.

### Topology analysis of augmented GIN

We assembled an augmented GIN using adjacency matrices^85^ comprising the 1660 hits in the 20 primary SGAs of this paper arising from the five probes (atorvastatin, cerivastatin, *hmg1-Δ, hmg2-Δ, arv1-Δ*) in the four genetic backgrounds (Supplementary Table S4) and additional genes from the published global GIN^12,86^ to include all genes less than two levels of distance apart (i.e., path length of 3) from the seed vertices of the genes from the 20 primary SGAs utilising *K*-edge centralities. The resulting 20 adjacency matrix files of ∼2000 × ∼2000 nodes each made up our 20 augmented GINs (Supplementary Table S6) were used for topology analyses in this paper (Figs. 4, 5). Closeness centrality, betweenness centrality and eigenvector centrality were calculated as previously described.^75^ Interactions were additionally filtered based on established stringent cut offs for digenic and trigenic interactions.^12^ Bootstrap analysis (*n* = 1000) of a random sample set of genes of the same size was statistically compared with our list using a Mann–Whitney rank test. Only networks with *P*-values below 0.05 were included in the topology analyses.

### Functional community clustering analysis

Networks were visualised as sorted adjacency matrices for best community modularity using the Louvain method.^46^ Defining *A* as edge weight, *k* as the sum of the weights of the edges attached to respective vertex, 2*m* as the sum of all of the edge weights in the graph, *C* as the community, and *δ* as Kronecker delta for a weighted network with vertices *i*, random walk modularity *M* was defined as:

$$M = \frac{1}{{2m}}\mathop {\sum }\limits_{ij} \left[ {A_{ij} - \frac{{k_ik_j}}{{2m}}} \right]\delta \left( {c_i,c_j} \right).$$

Change of modularity after reassigning the community was calculated as follows:

$$\Delta M = \left[ {\left( {\frac{{\mathop {\sum }\nolimits_{in} \,+\, 2\,k_{ijn}}}{{2m}} - \frac{{\mathop {\sum }\nolimits_{{\mathrm {tot}}} \,+\, k_j}}{{2m}}} \right)^2} \right] - \left[ {\frac{{\mathop {\sum }\nolimits^ in}}{{2m}} - \left( {\frac{{\mathop {\sum }\nolimits_{{\mathrm {tot}}} }}{{2m}}} \right)^2 - \left( {\frac{{k_j}}{{2m}}} \right)^2} \right].$$

### GO enrichment analysis

GO terms were identified using GOATOOLS^48^ for each community and statistically evaluated using *P*-values that were corrected using a 2-stage Benjamini–Krieger–Yekutieli false discovery rate (FDR). Distinct, functional communities were defined by ‘uniqueness’, a score that represents the negative of average semantic similarity of a term to all other terms according to Lin’s semantic similarity.^87^

### UPR analysis

The UPR was evaluated using a highly specific fluorescent marker as previously described.^38,39^ Strains expressing four tandem repeats of the UPR elements fused to a GFP (4X-UPRE-GFP) were subcultured to an OD of 0.2 and treated with appropriate IC_20_ concentrations of atorvastatin or cerivastatin for 4 h at 30 °C. Fluorescence was measured using a FACSantoTM II flow cytometer (Becton Dickinson) across 100,000 total events. Cells stained with 4 µg/mL of propidium iodide (PI) to determine cell viability were first identified by gating on FSC-A/SSC-A. Next, a singlets gate was placed on single cells by SSC-A/SSC-W. Singlets were then assessed by FSC-A against PI fluorescence using the 488 laser and 585/42 filter set, and a live cells gate placed on the PI-negative population (Supplementary Fig. S18). GFP expression was quantified in the whole live-cell population using the 488 laser and 530/30 filter set, and data presented as the geometric mean fluorescence intensity ± median absolute deviation was calculated using FloJo (Becton Dickinson).

### Reporting summary

Further information on experimental design is available in the Nature Research Reporting Summary linked to this paper.

## Supplementary information

Supplementary Table 1

Supplementary Table 2

Supplementary Table 3

Supplementary Table 4

Supplementary Table 5

Supplementary Table 6

Supplementary Table 7

Supplementary Table 8

Supplementary Table 9

Supplementary Table 10

Supplementary Table 11

Supplementary Table 12

Supplementary Table Legends

Supplementary Figures

Nature research reporting summary

## Data Availability

The datasets for this article are available as part of the Supplementary Information. Raw genome sequence data from the three new ssDMAs (Y55, UWOPS87-2421, YPS606) are publically available at SRA number SRP124399 and BioProject PRJNA417352.
