# Publisher Correction: Genetic interaction networks mediate individual statin drug response in *Saccharomyces cerevisiae*

**DOI:** 10.1038/s41540-020-0129-9

**Published:** 2020-04-02

**Authors:** Bede P. Busby, Eliatan Niktab, Christina A. Roberts, Jeffrey P. Sheridan, Namal V. Coorey, Dinindu S. Senanayake, Lisa M. Connor, Andrew B. Munkacsi, Paul H. Atkinson

**Affiliations:** 1 Centre for Biodiscovery, School of Biological Sciences, Victoria University of Wellington, Wellington, New Zealand; 2 European Molecular Biology Laboratory, Meyerhofstraße 1, 69117 Heidelberg, Germany

**Keywords:** Epistasis, Biochemical networks, Screening, Epistasis

Correction to: *npj Systems Biology and Applications* 10.1038/s41540-019-0112-5, published online 3 October 2019.

The Supplementary Tables 1–12 were missing from the Article. These have now been added.

