# Supplementary material for: Genetic interaction networks mediate individual statin drug response in Saccharomyces cerevisiae
Source: NPJ Syst Biol Appl. 2019 Oct 3;5:35. doi: 10.1038/s41540-019-0112-5 (PMC6776536; doi:10.1038/s41540-019-0112-5)
Supplement: Supplementary file 13 — Supplementary Table Legends [file 41540_2019_112_MOESM13_ESM.pdf]

Supplementary Table S1. Yeast strains used in this study.

Supplementary Table S2. Pairwise alignment of ssDMA libraries to the corresponding SGRP and S288C parental strains.

Supplementary Table S3. Linkage groups in SGA analyses.

Supplementary Table S4. Genetic interaction scores for the 20 primary GINs.

Supplementary Table S5. Adjacency matrix files of ~2000 x ~2000 nodes for 20 augmented GINs.

Supplementary Table S6. Data used for the SAFE analysis.

Supplementary Table S7. Community analysis GO terms by coverage and score.

Supplementary Table S8. Community analysis genes.

Supplementary Table S9. Cluster Silhouette and Calinski-Harabasz Index scores.

Supplementary Table S10. Uniqueness scores.

Supplementary Table S11. Primers used in this study.

Supplementary Table S12. Plasmids used in this study.
