# Supplementary material for: Genetic interaction networks mediate individual statin drug response in Saccharomyces cerevisiae
Source: NPJ Syst Biol Appl. 2019 Oct 3;5:35. doi: 10.1038/s41540-019-0112-5 (PMC6776536; doi:10.1038/s41540-019-0112-5)
Supplement: Supplementary file 14 — Supplementary Figures [file 41540_2019_112_MOESM14_ESM.pdf]

# Supplementary Figure S1

a

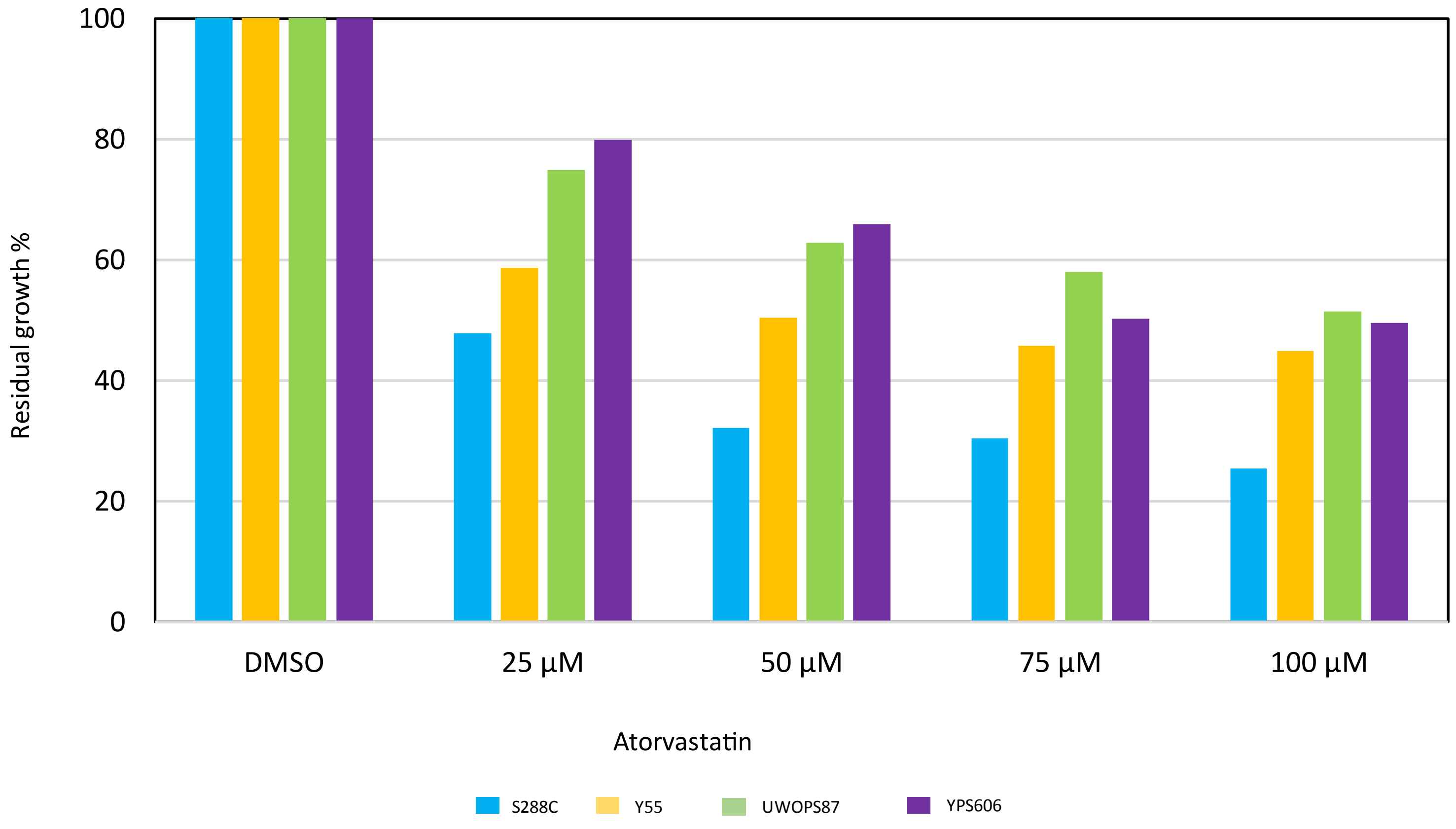


b

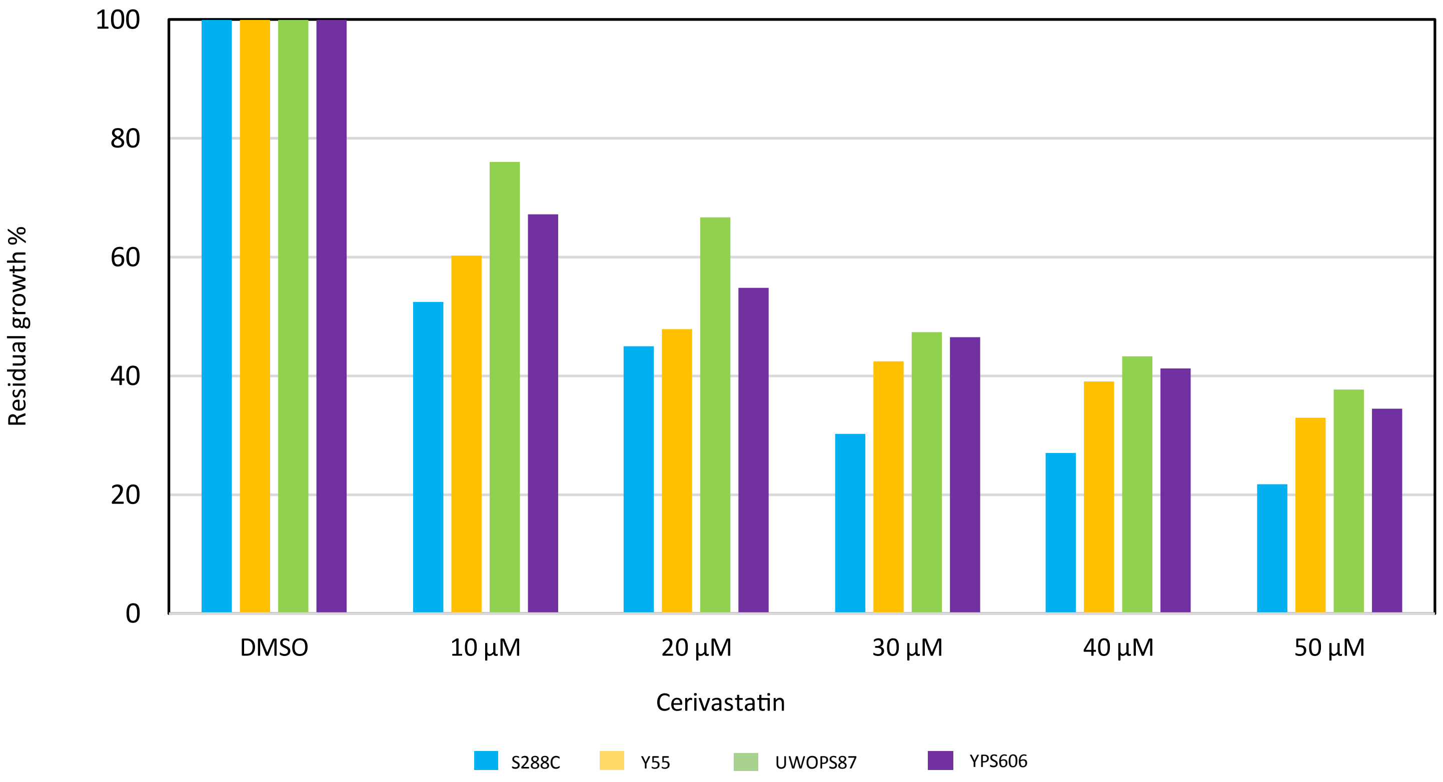


**Supplementary Figure S1**. Residual growth (a ratio of growth in treated and untreated cells) of all 1536 colony size values from plate 10 of the original S288C DMA compared to the three newly created ssDMA libraries in the genetic backgrounds of Y55, UWOPS87-2421 (UWOPS87) and YPS606. Growth was quantified in a) atorvastatin and b) cerivastatin.

## Supplementary Figure S2

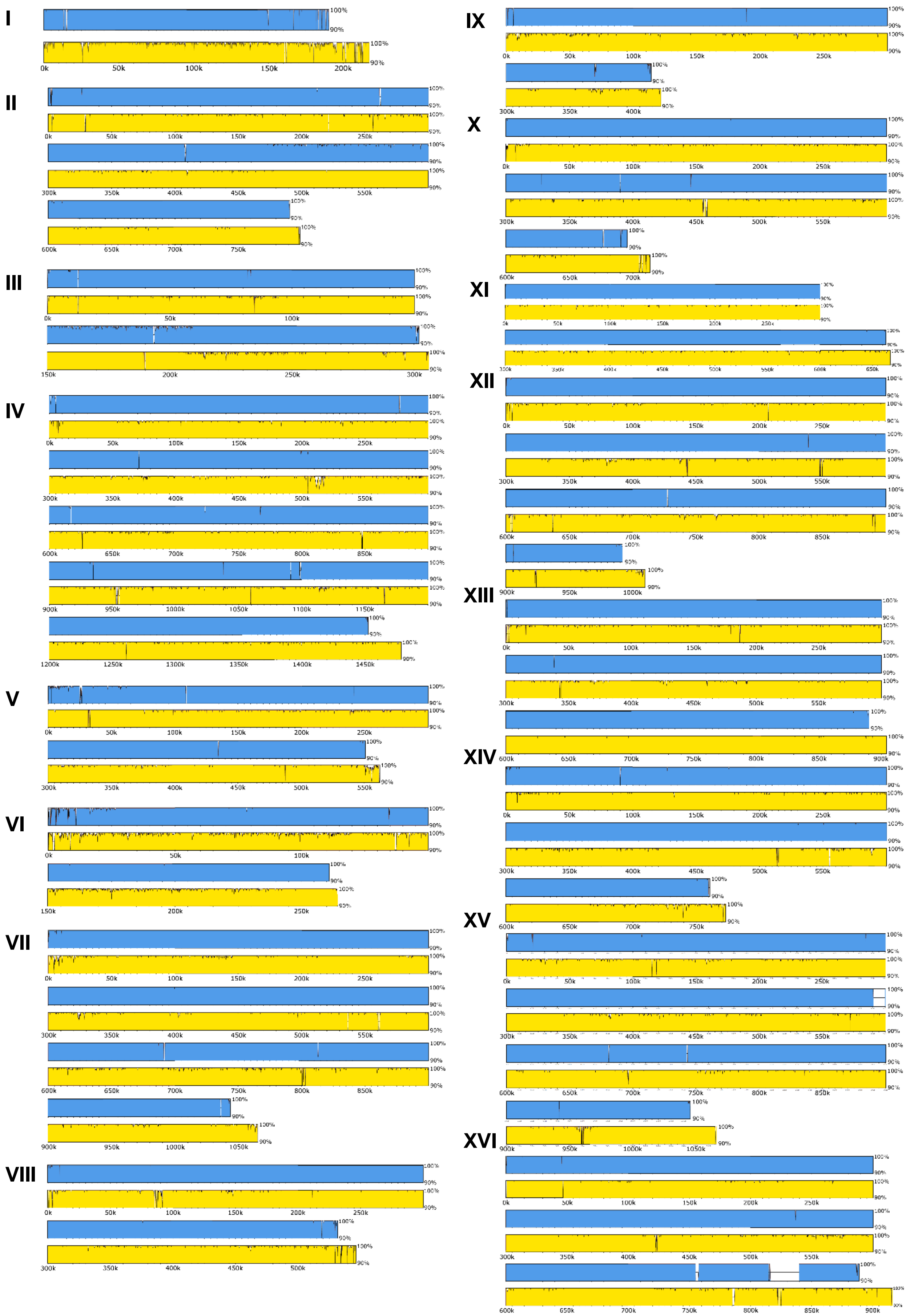


**Supplementary Figure S2**. UWOPS87-2421 ssDMA alignment to UWOPS87-2421 parental strain (blue) and S288C parental strain (yellow).Chromosomes I-XVI

# Supplementary Figure S3

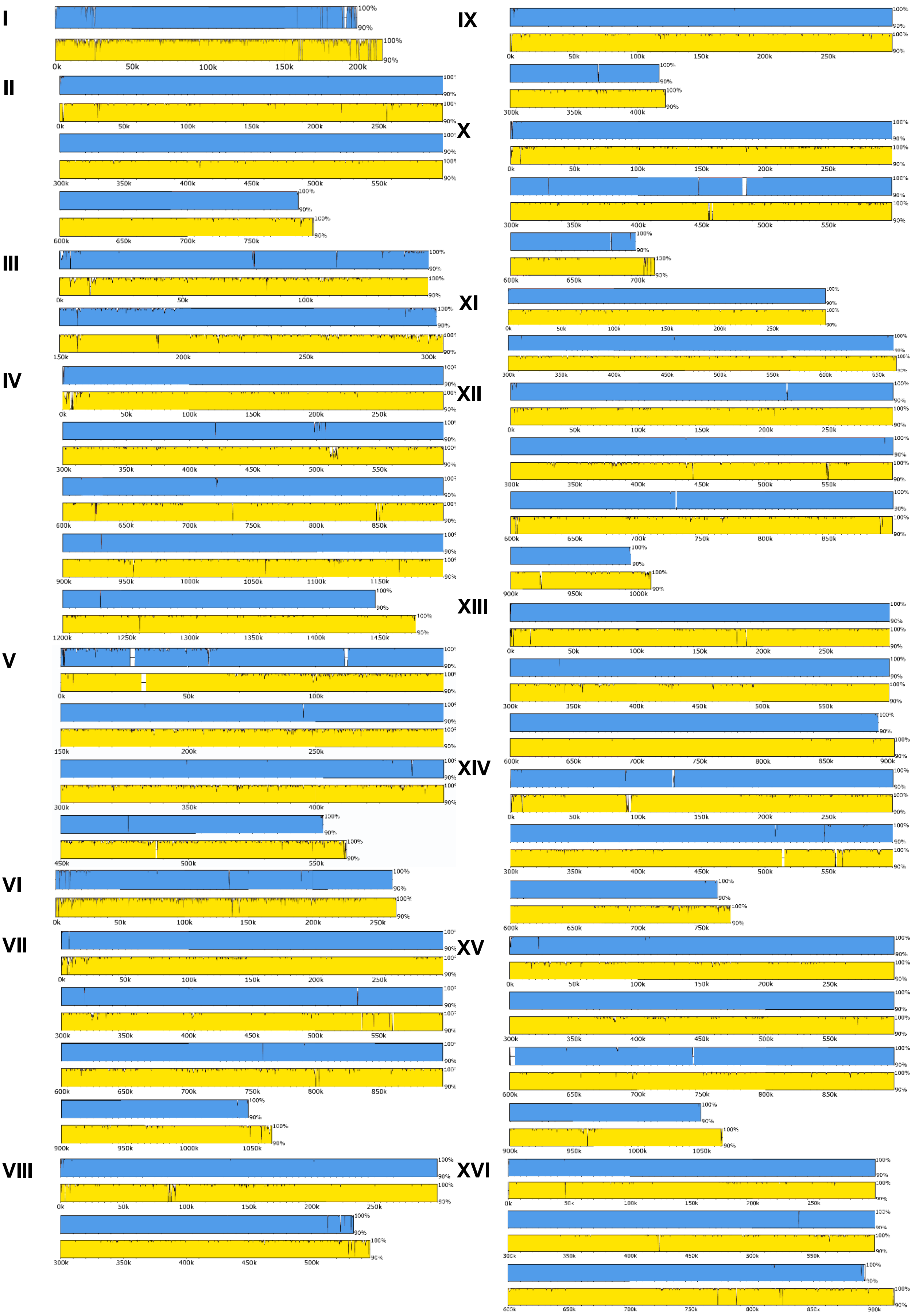


**Supplementary Figure S3**. Y55 ssDMA alignment to Y55 parental strain (blue) and S288C parental strain (yellow). Chromosomes I-XVI

# Supplementary Figure S4

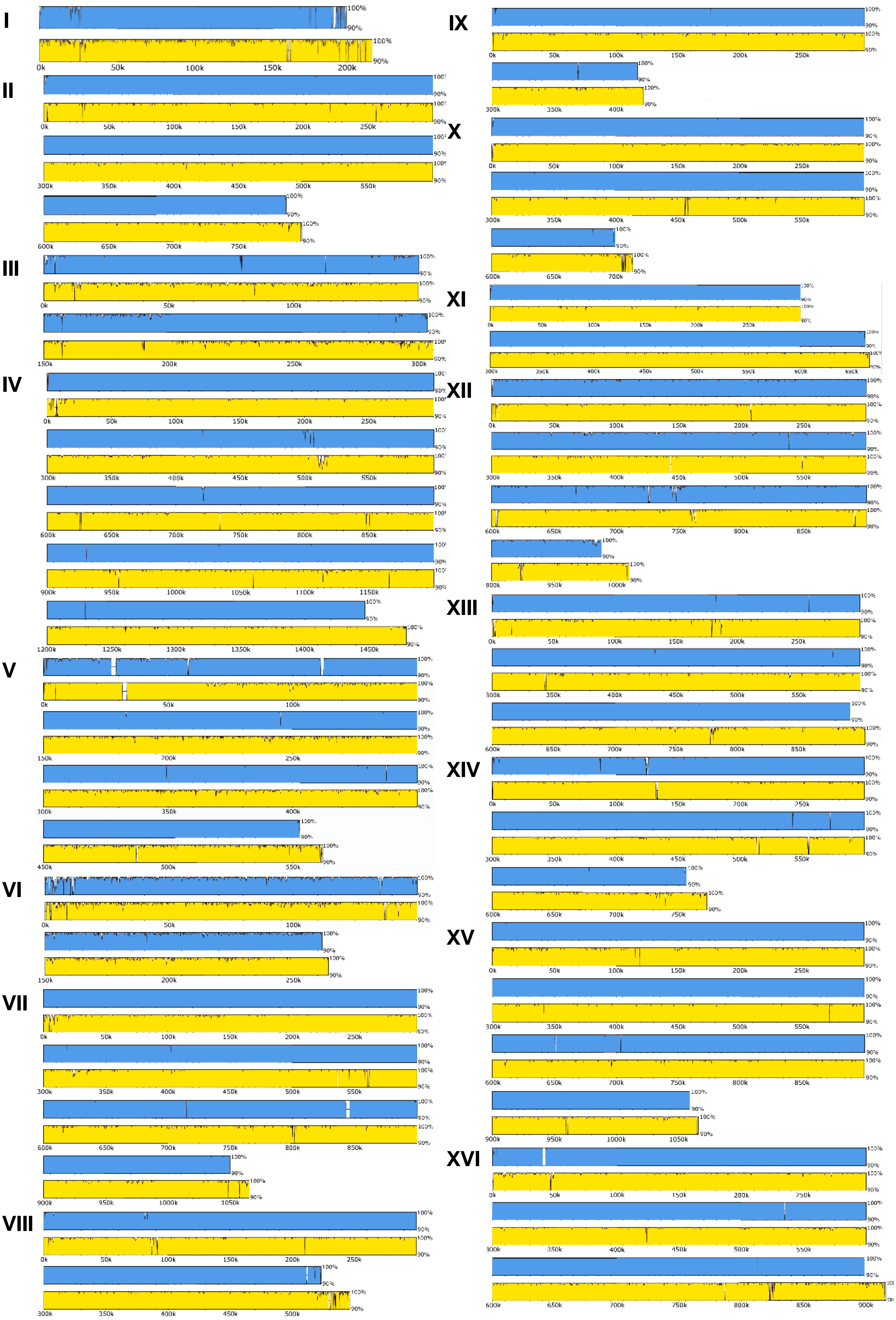


**Supplementary Figure S4.** YPS606 ssDMA alignment to YPS606 parental strain (blue) and S288C parental strain (yellow). Chromosomes I-XVI

## Supplementary Figure S5

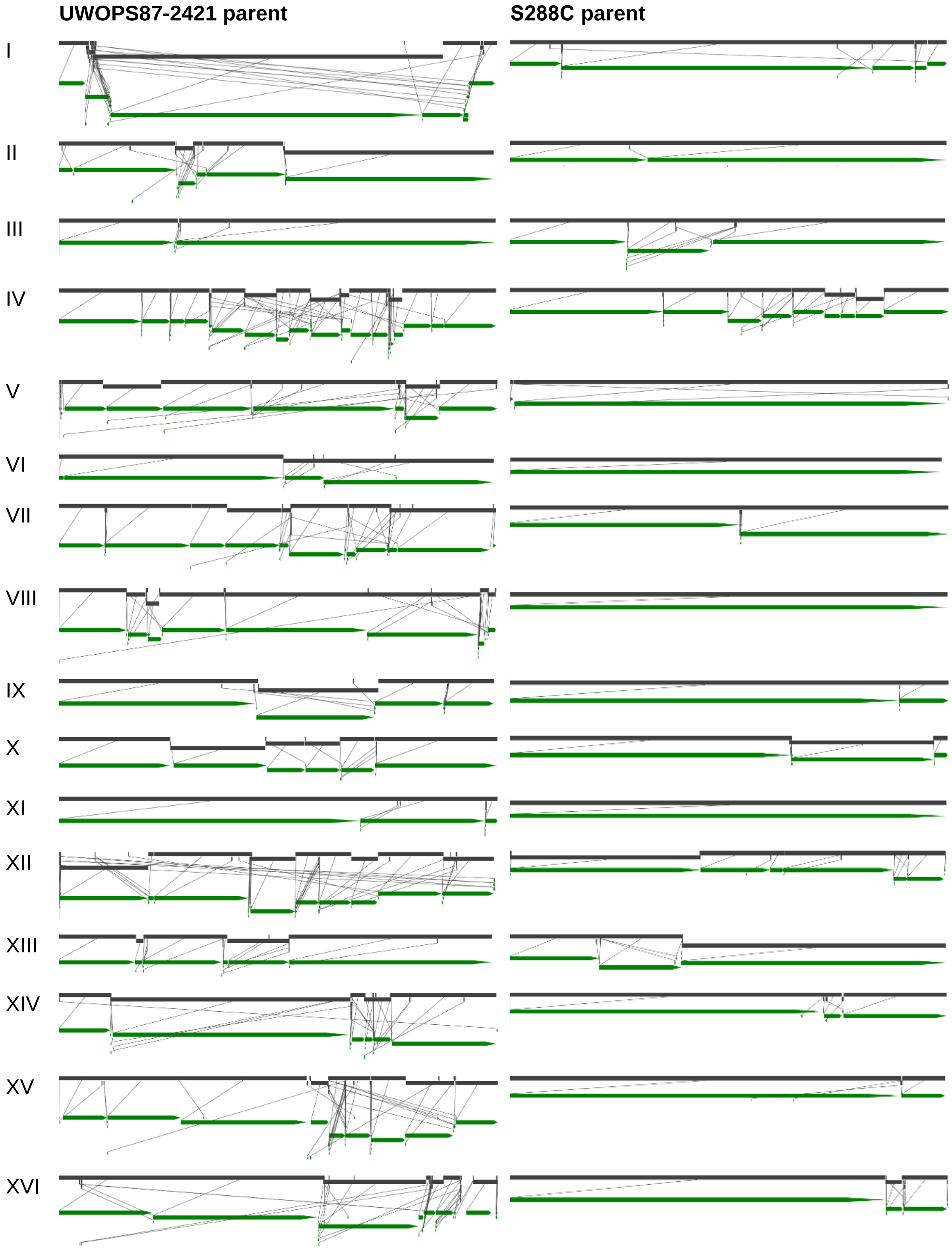


**Supplementary Figure S5.** Synteny between UWOPS87-2421 ssDMA (grey) and its respective UWOPS87-2421 and S288C parental strains (green). Chromosomes **I-XVI**

# Supplementary Figure S6

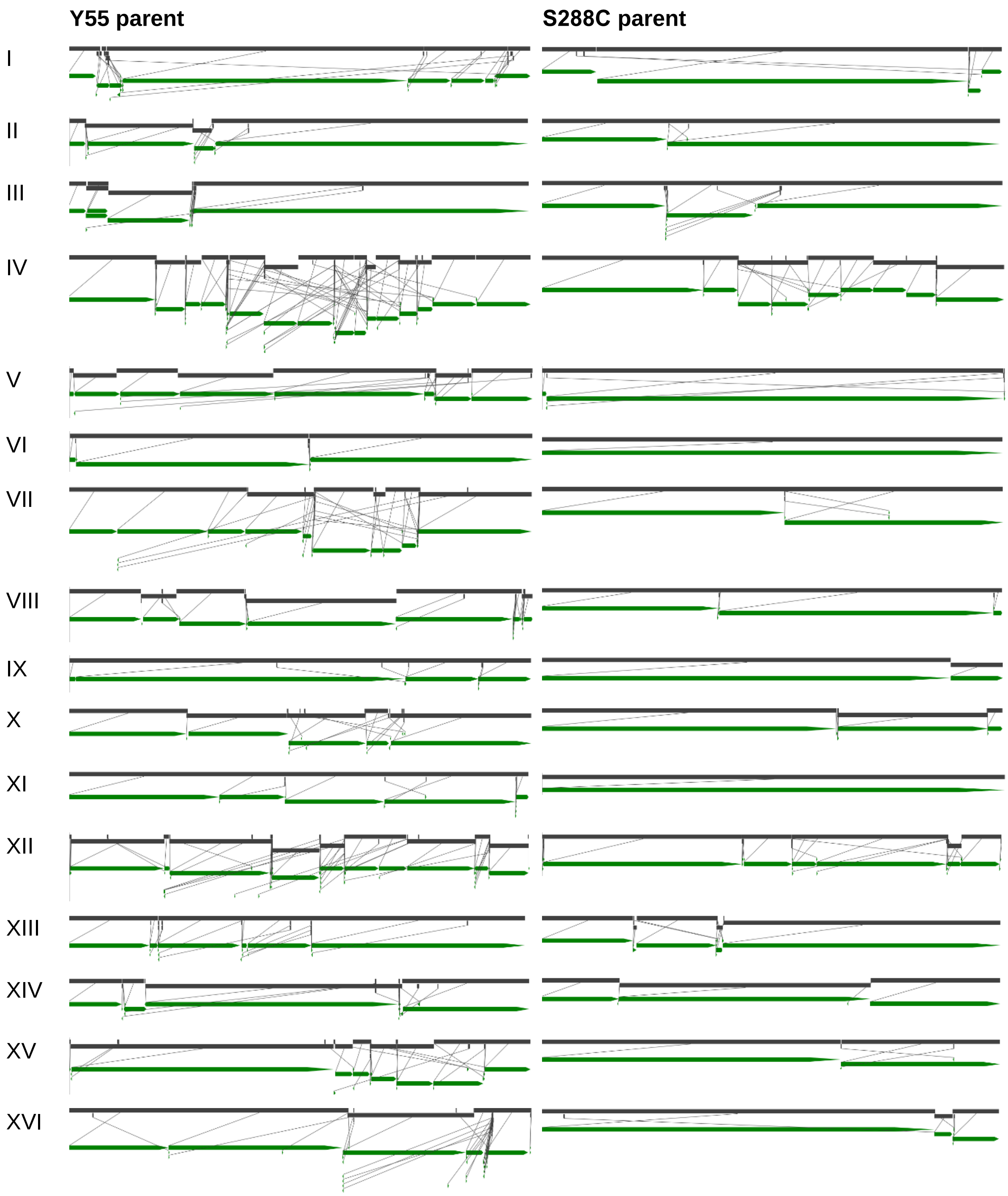


**Supplementary Figure S6.** Synteny between Y55 ssDMA (grey) and its respective Y55 and S288C parental strains (green). Chromosomes I-XVI

# Supplementary Figure S7

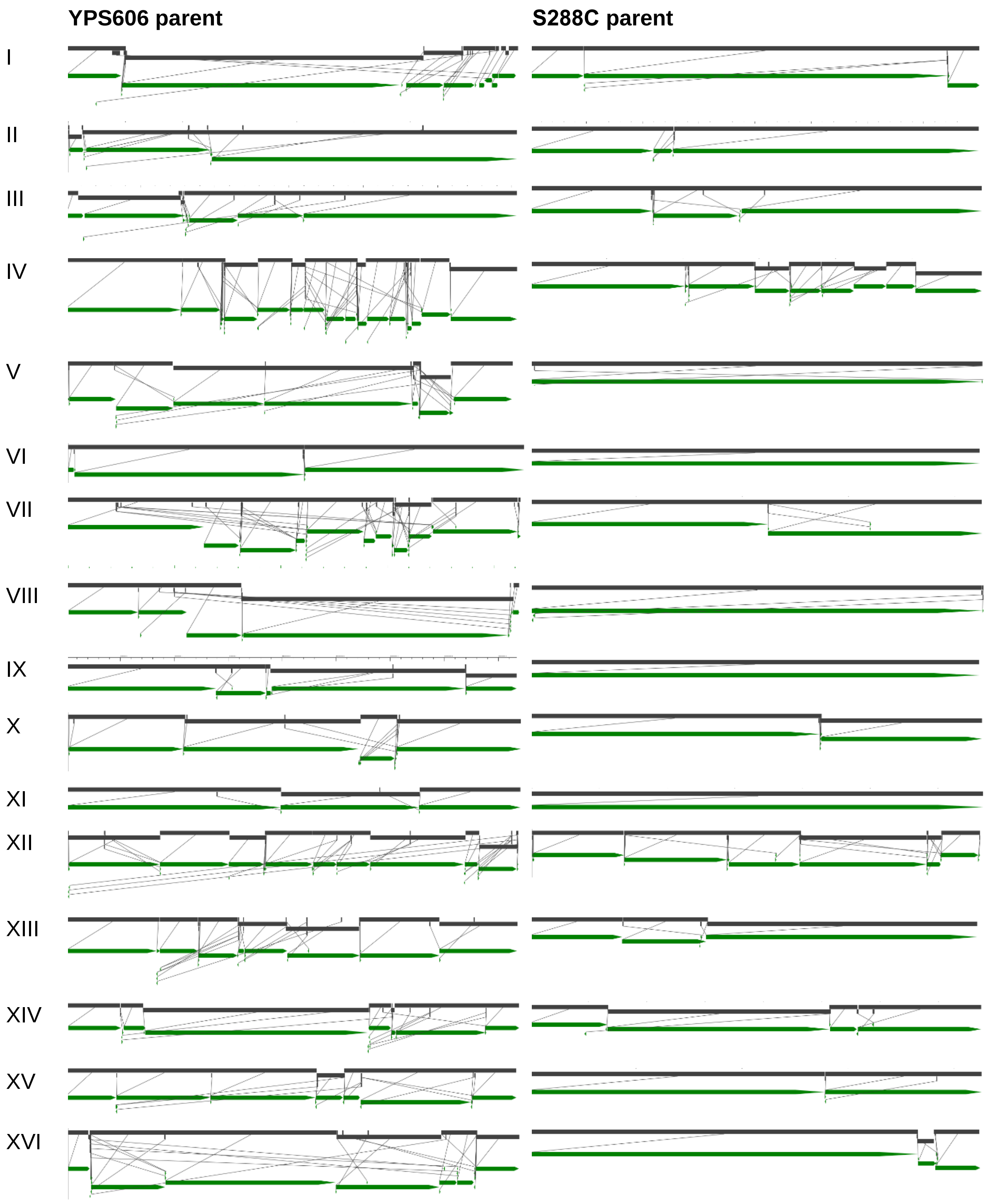


**Supplementary Figure S7.** Synteny between YPS606 ssDMA (grey) and its respective S288C and S288C parental strains (green). Chromosomes I-XVI

## Supplementary Figure S8

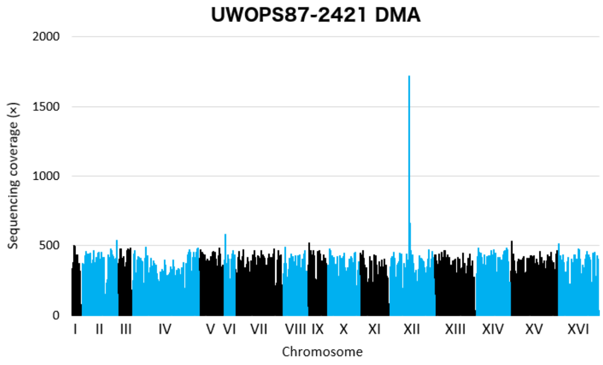


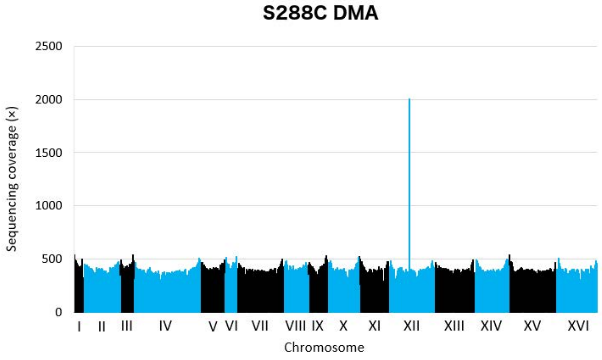


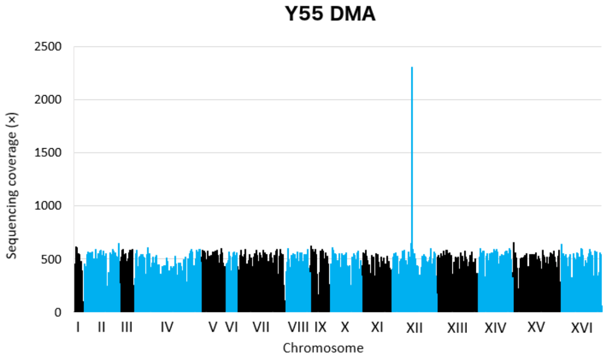


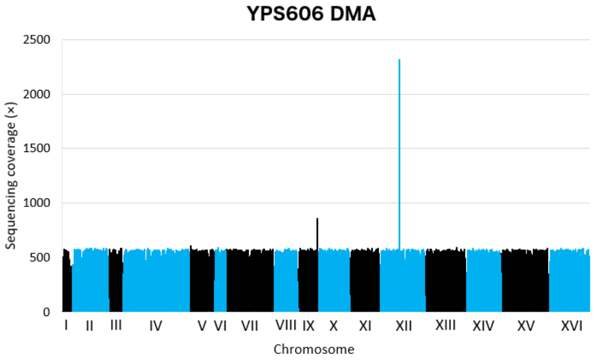


**Supplementary Figure S8.** Sequencing coverage of the ssDMA libraries (UWOPS87-2421, Y55, YPS606) and the control S288C DMA library shows no evidence of chromosomal aneuploidy.

## Supplementary figure S9

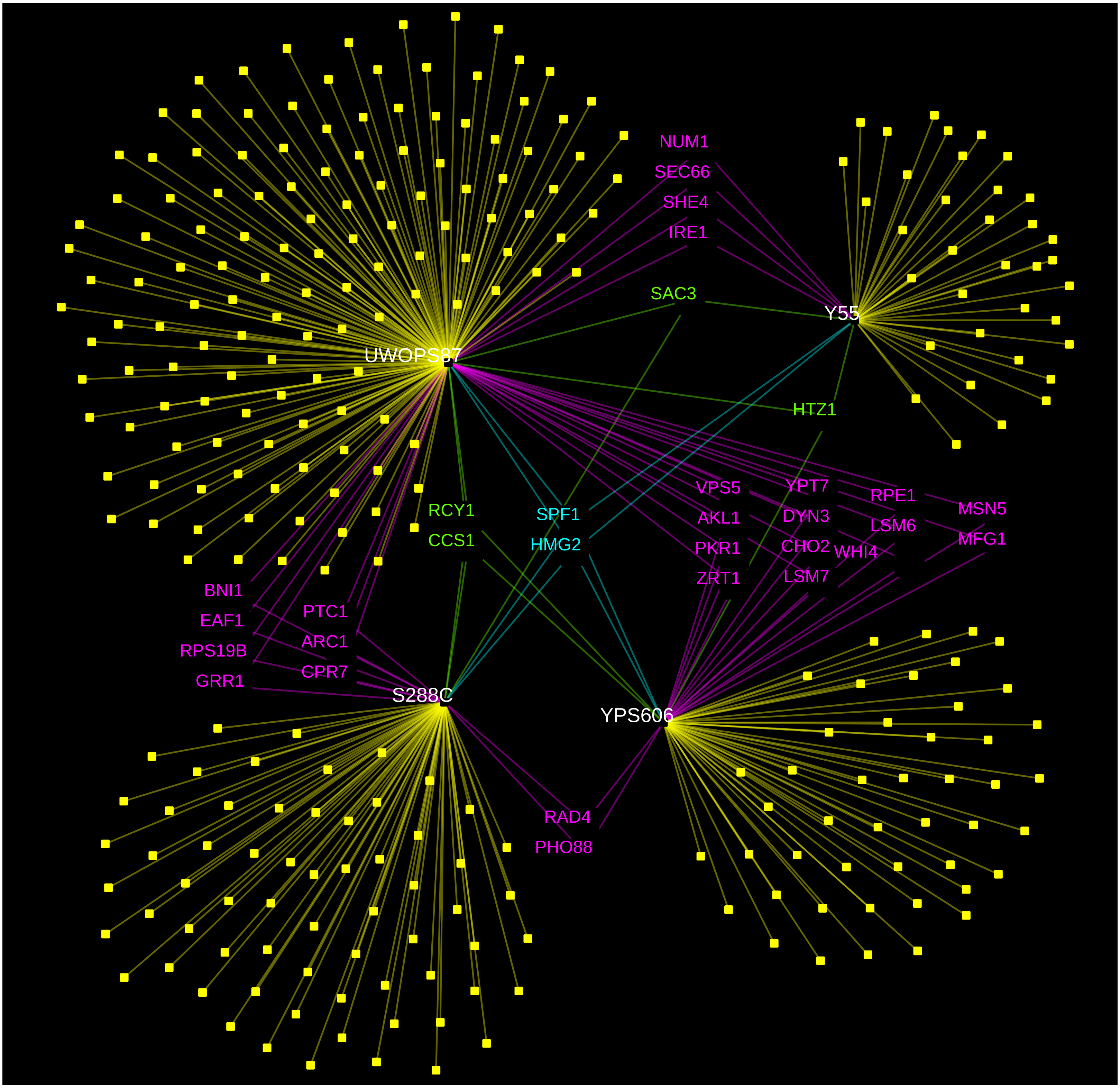


**Supplementary Figure S9.** Primary genetic interaction network showing HMG1 interactions in the four genetic background deletion libraries (S288C, Y55, UWOPS87-2421 (UWOPS87) and YPS606). Interactions are shared by different genetic backgrounds (purple, green, and blue) or unique to specific genetic backgrounds (yellow, genes not included in this figure).

**Supplementary figure S10**

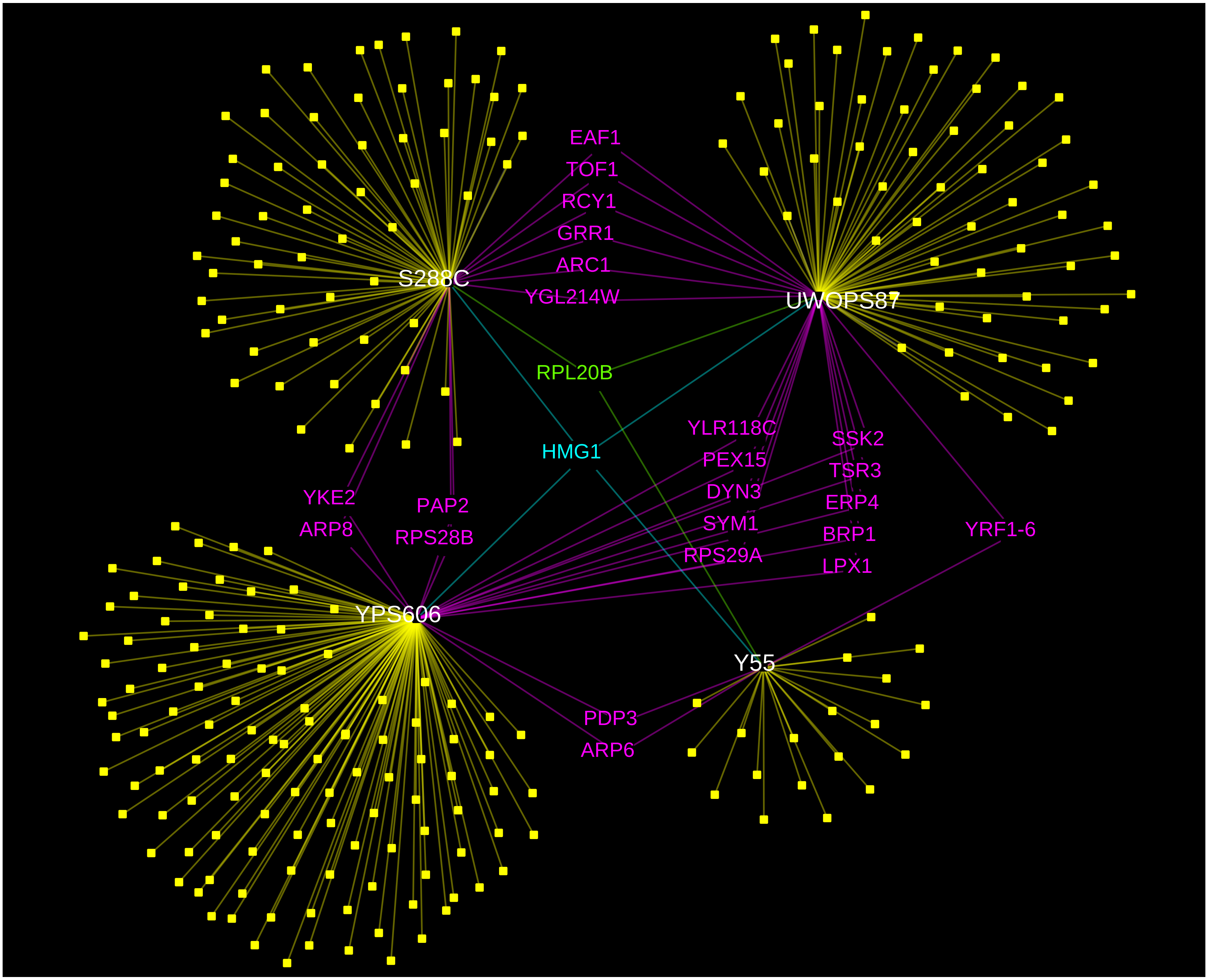


**Supplementary Figure S10.** Primary genetic interaction network showing HMG2 interactions in the four genetic background deletion libraries (S288C, Y55, UWOPS87-2421 (UWOPS87) and YPS606). Interactions are shared by different genetic backgrounds (purple, green, and blue) or unique to specific genetic backgrounds (yellow, genes not included in this figure).

## Supplementary figure S11

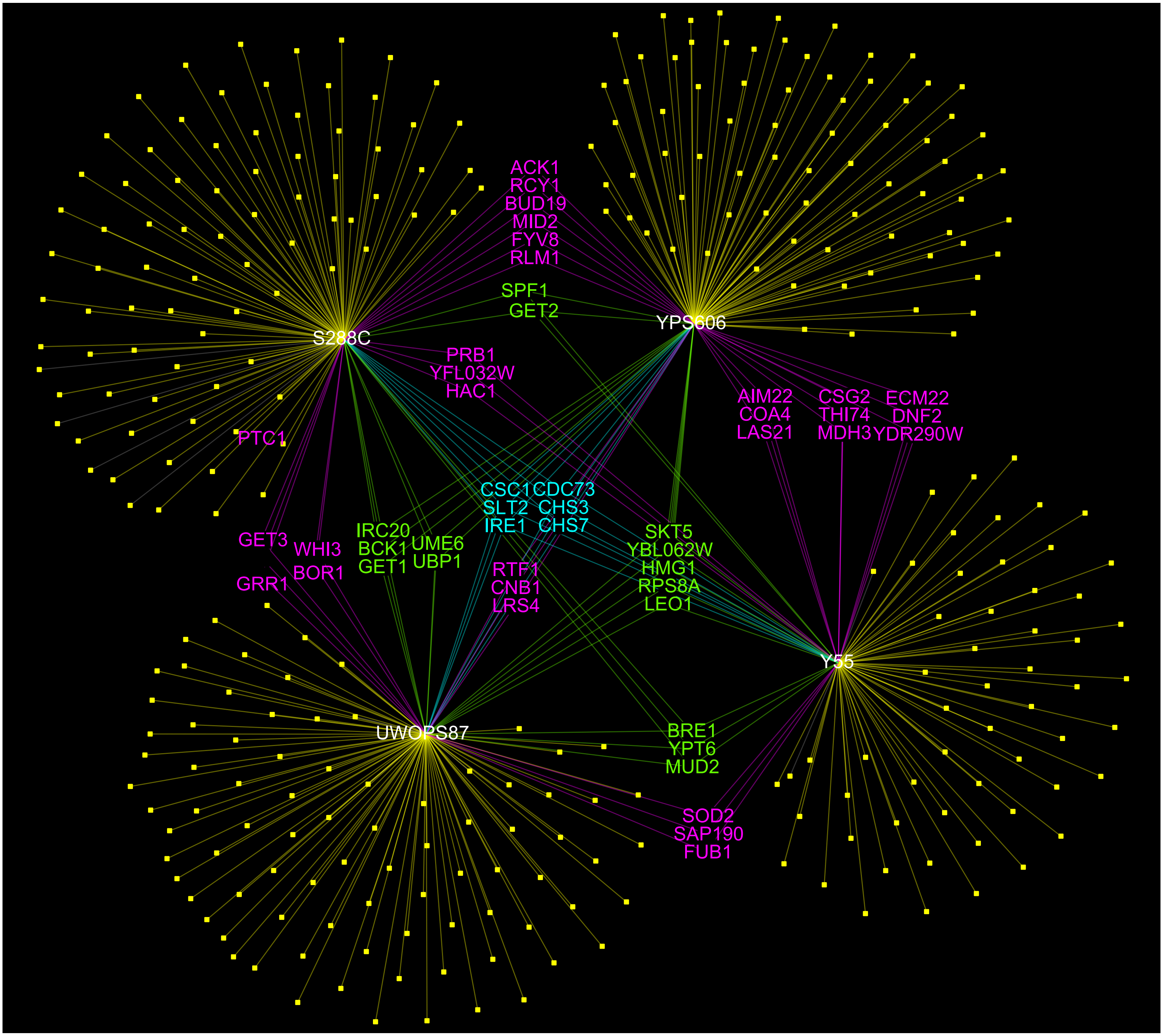


**Supplementary Figure S11.** Primary genetic interaction network showing ARV1 interactions in the four genetic background deletion libraries (S288C, Y55, UWOPS87-2421 and YPS606). Interactions are shared by different genetic backgrounds (purple, green, and blue) or unique to specific genetic backgrounds (yellow, genes not included in this figure).

## Supplementary figure S12

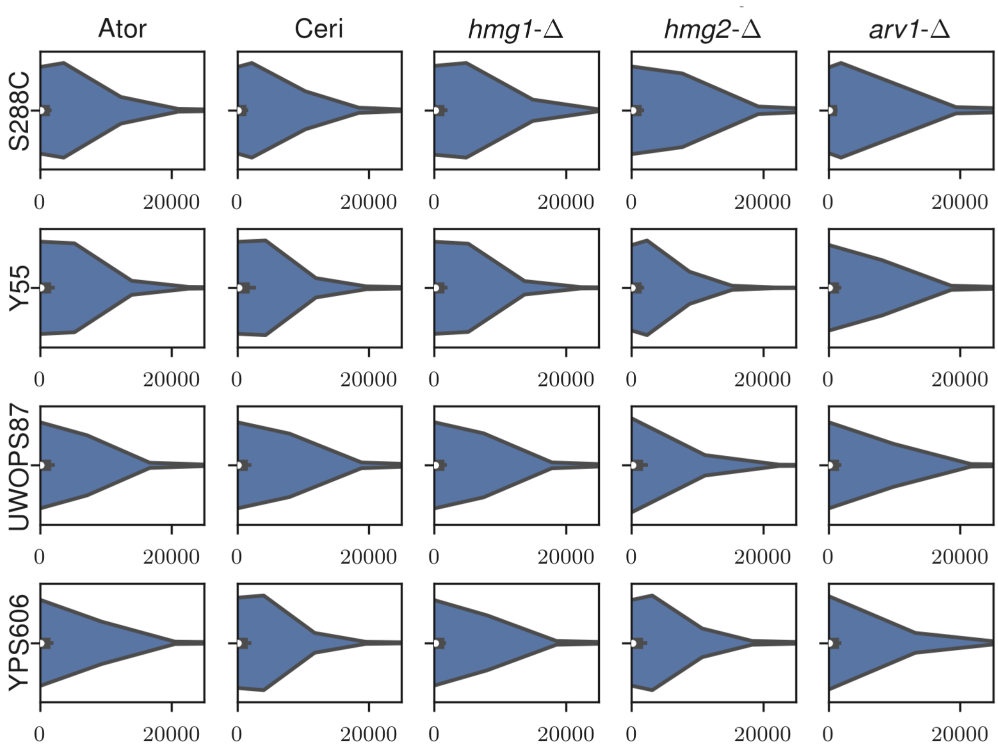


**Supplementary Figure S12.** Distribution of gene betweenness centrality values for GIs in augmented networks of four strains (S288C, Y55, UWOPS87-2421 (UWOPS87) and YPS606) by five queries (atorvastatin, cerivastatin, hmg1-Δ, hmg2-Δ and arv1-Δ).

## Supplementary figure S13

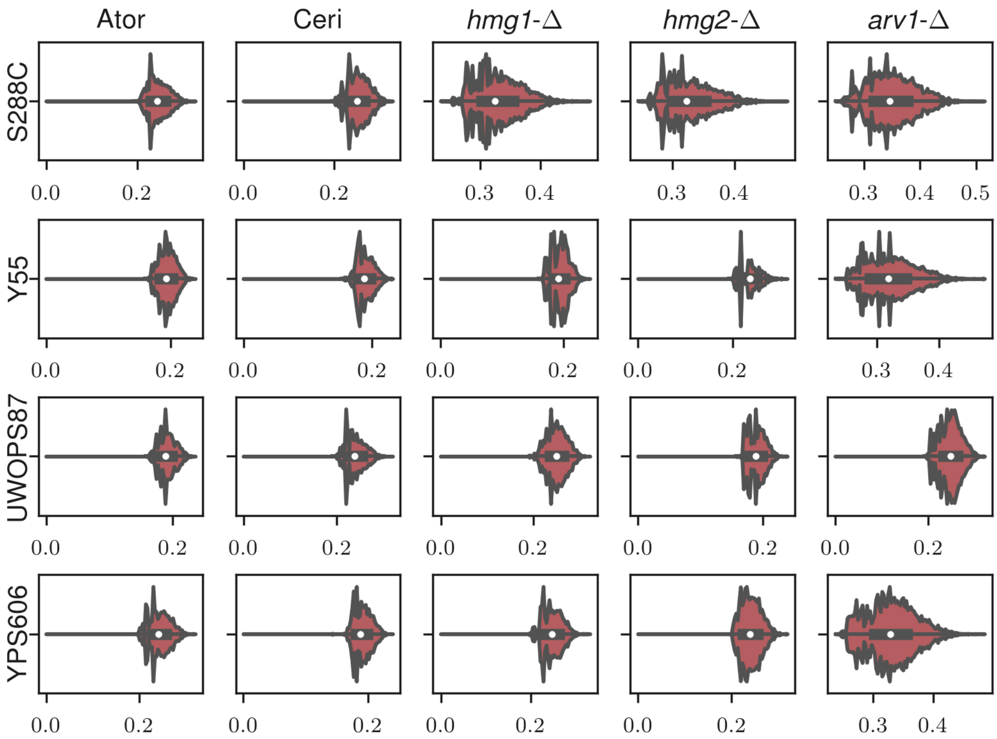


**Supplementary Figure S13**. Distribution of gene closeness centrality values for GIs from in augmented networks of four strains (S288C, Y55, UWOPS87-2421 (UWOPS87) and YPS606) by five queries (atorvastatin, cerivastatin, hmg1-Δ, hmg2-Δ and arv1-Δ).

## Supplementary figure S14

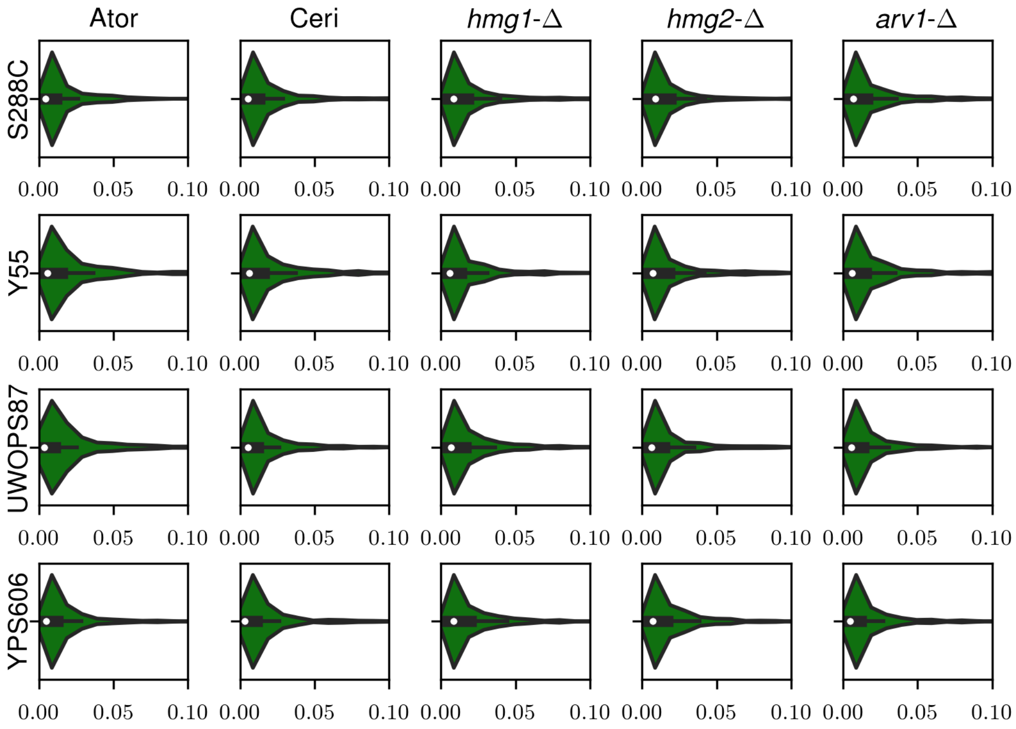


**Supplementary Figure S14.** Distribution of gene eigenvector centrality values for GIs in augmented networks of four strains (S288C, Y55, UWOPS87-2421 (UWOPS87) and YPS606) by five queries (atorvastatin, cerivastatin, hmg1-Δ, hmg2-Δ and arv1-Δ).

# Supplementary figure S15

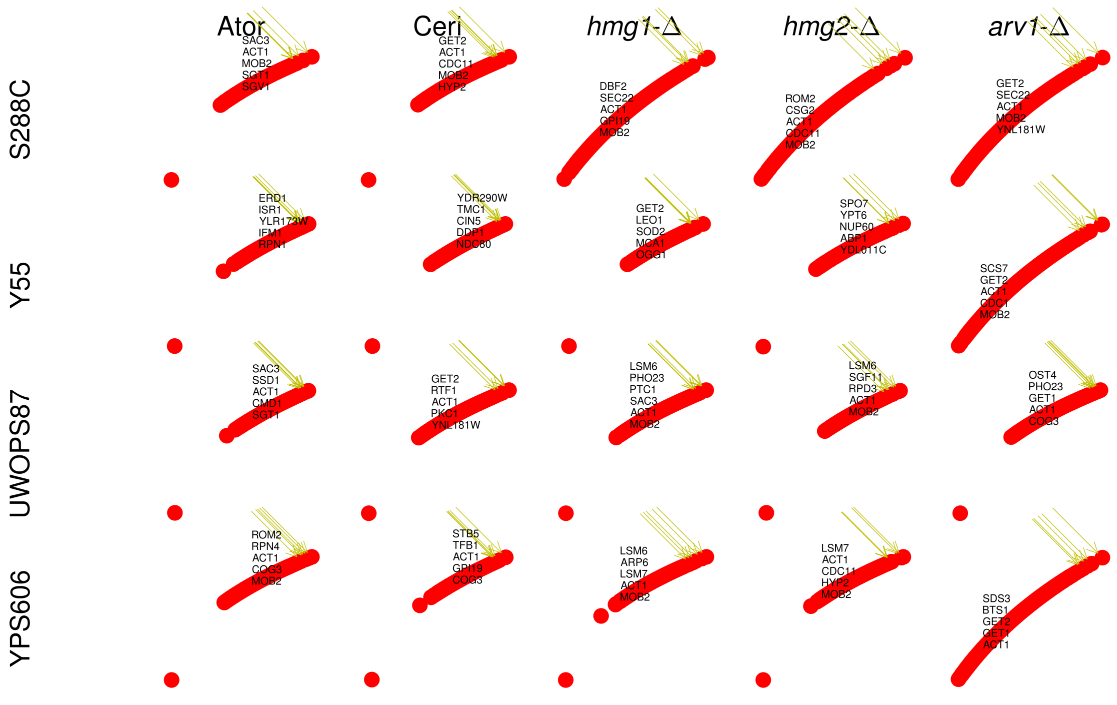


**Supplementary Figure S15.** Genes with maximum closeness centrality deconvoluted from distribution data shown in Supplementary Figure S13 in four strains (S288C, Y55, UWOPS87-2421 (UWOPS87) and YPS606) by five queries (atorvastatin, cerivastatin, hmg1-Δ, hmg2-Δ and arv1-Δ).

## Supplementary figure S16

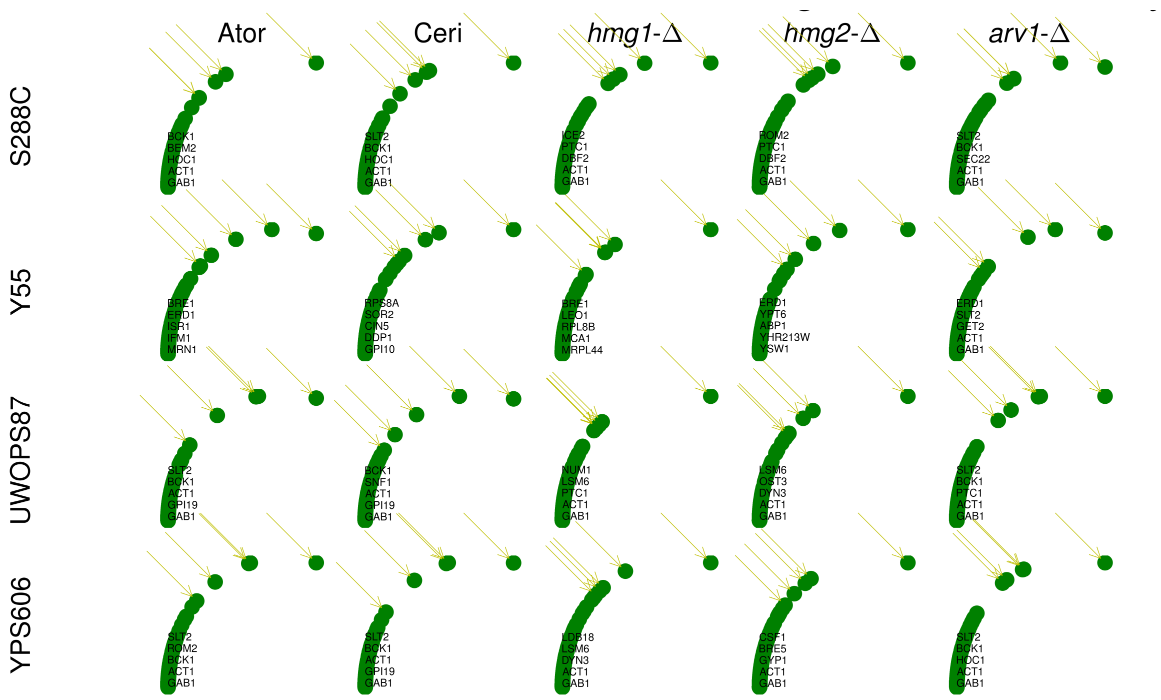


**Supplementary Figure S16.** Genes with maximum eigenvector centrality deconvoluted from distribution data shown in Supplementary Figure S14 in four strains (S288C, Y55, UWOPS87-2421 (UWOPS87) and YPS606) by five queries (atorvastatin, cerivastatin, hmg1-Δ, hmg2-Δ and arv1-Δ).

**Supplementary figure S17**

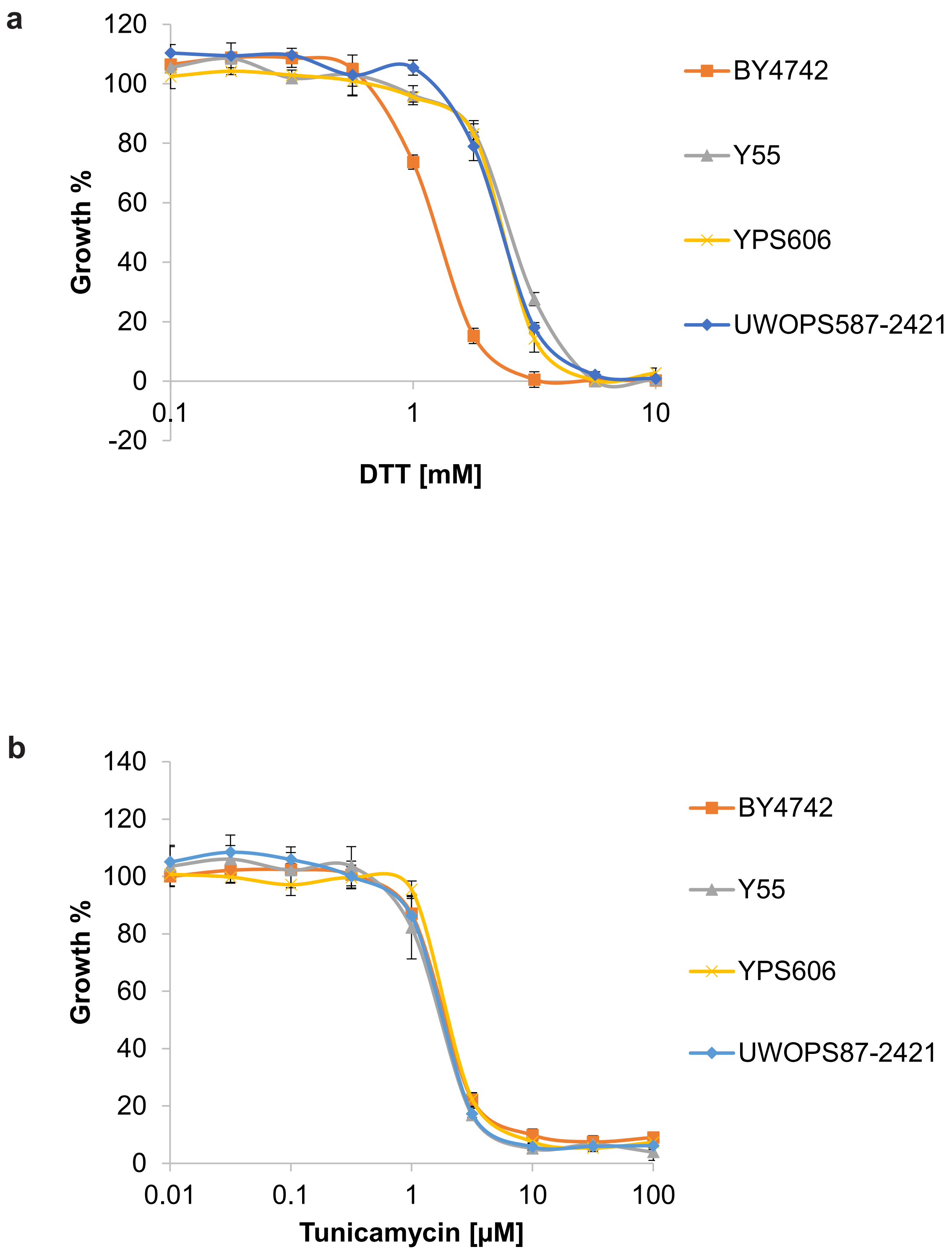


**Supplementary Figure S17**. Robustness of strains to growth in DTT or TM. a) % growth of S288C, Y55, YPS606 and UWOP587 treated with dithiothreitol (DTT) relative to carrier dH2O. b) % growth of S288C, Y55, YPS606 and UWOP587 strains treated with tunicamycin (TM) relative to carrier DMSO. Yeast strains cultured overnight in SC broth were diluted to 5x105 cells/mL in SC broth and treated with DTT, TM or carrier DMSO or dH2O in 96 well microtiter plate. Percent growth under drug treatment was quantified relative to the carrier (i.e. OD590TM / OD590DMSO x 100) from triplicate experiments, error bars are standard deviation.

**Supplementary figure S18**

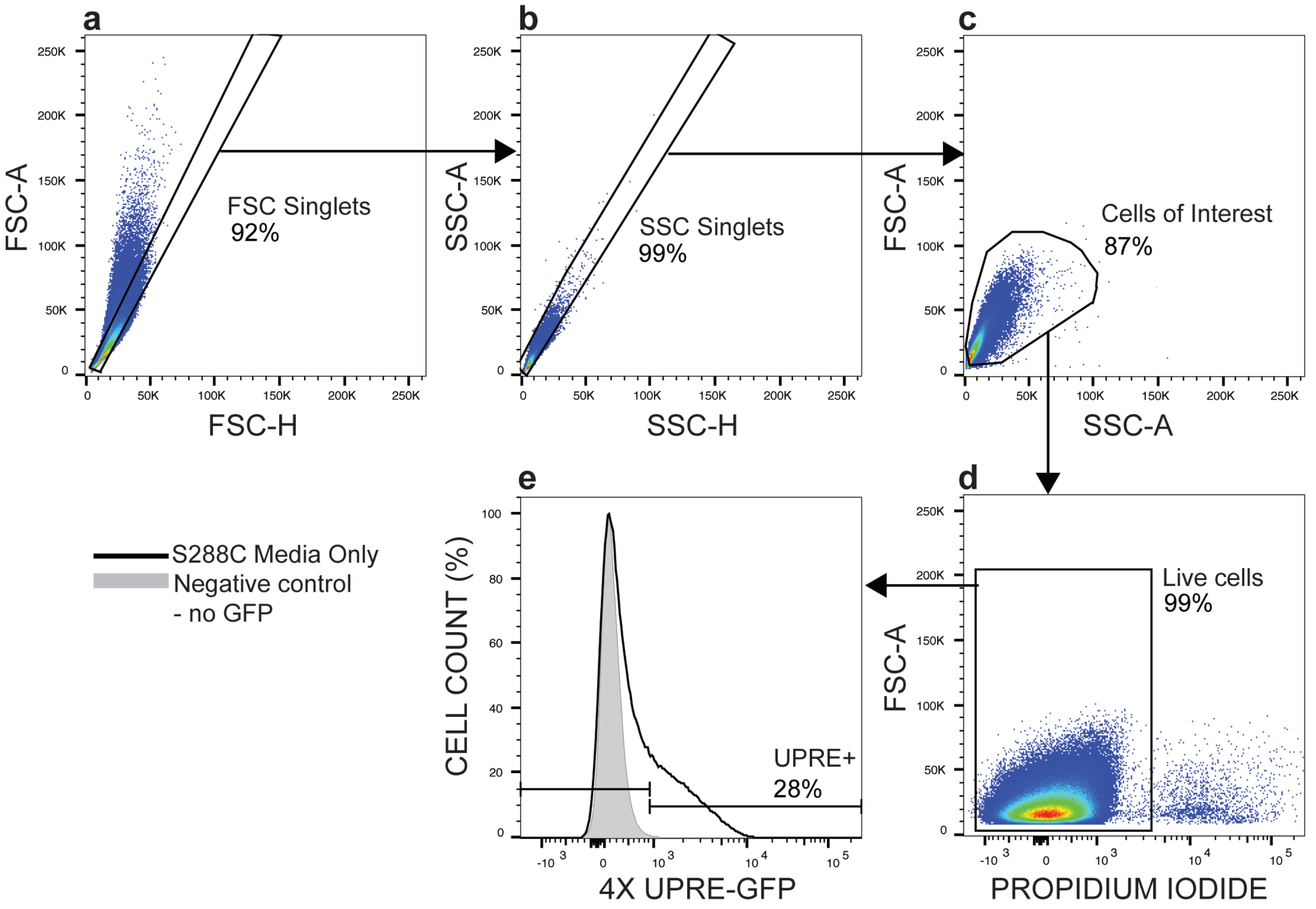


**Supplementary Figure S18.** Gating Strategy for 4XUPRE GFP in yeast cells. Flow cytometry analysis was performed on 4XUPRE GFP expressing yeast strains as described in Figure 6. Pseudocolour plots indicate forward scatter (FSC), a) and side scatter (SSC), b) doublet exclusion, cells of interest c) and live cells d). Histogram in e) shows percent GFP expression on live, non-GFP expressing yeast cells (solid grey) and S288C yeast cells (black line).
